# Current Evidence for a Role of the Kynurenine Pathway of Tryptophan Metabolism in Multiple Sclerosis

**DOI:** 10.3389/fimmu.2016.00246

**Published:** 2016-08-04

**Authors:** Michael D. Lovelace, Bianca Varney, Gayathri Sundaram, Nunzio F. Franco, Mei Li Ng, Saparna Pai, Chai K. Lim, Gilles J. Guillemin, Bruce J. Brew

**Affiliations:** ^1^Applied Neurosciences Program, Peter Duncan Neurosciences Research Unit, St Vincent’s Centre for Applied Medical Research, Sydney, NSW, Australia; ^2^Faculty of Medicine, St Vincent’s Clinical School, University of New South Wales, Sydney, NSW, Australia; ^3^Faculty of Medicine, Sydney Medical School, University of Sydney, Sydney, NSW, Australia; ^4^Sydney Medical School, University of Sydney, Sydney, NSW, Australia; ^5^Neuroinflammation Group, Faculty of Medicine and Health Sciences, Macquarie University, Sydney, NSW, Australia; ^6^Department of Neurology, St Vincent’s Hospital, Sydney, NSW, Australia

**Keywords:** kynurenine pathway, neuroinflammation, neurodegenerative disease, multiple sclerosis, multiphoton microscopy

## Abstract

The kynurenine pathway (KP) is the major metabolic pathway of the essential amino acid tryptophan (TRP). Stimulation by inflammatory molecules, such as interferon-γ (IFN-γ), is the trigger for induction of the KP, driving a complex cascade of production of both neuroprotective and neurotoxic metabolites, and in turn, regulation of the immune response and responses of brain cells to the KP metabolites. Consequently, substantial evidence has accumulated over the past couple of decades that dysregulation of the KP and the production of neurotoxic metabolites are associated with many neuroinflammatory and neurodegenerative diseases, including Parkinson’s disease, AIDS-related dementia, motor neurone disease, schizophrenia, Huntington’s disease, and brain cancers. In the past decade, evidence of the link between the KP and multiple sclerosis (MS) has rapidly grown and has implicated the KP in MS pathogenesis. KP enzymes, indoleamine 2,3-dioxygenase (IDO-1) and tryptophan dioxygenase (highest expression in hepatic cells), are the principal enzymes triggering activation of the KP to produce kynurenine from TRP. This is in preference to other routes such as serotonin and melatonin production. In neurological disease, degradation of the blood–brain barrier, even if transient, allows the entry of blood monocytes into the brain parenchyma. Similar to microglia and macrophages, these cells are highly responsive to IFN-γ, which upregulates the expression of enzymes, including IDO-1, producing neurotoxic KP metabolites such as quinolinic acid. These metabolites circulate systemically or are released locally in the brain and can contribute to the excitotoxic death of oligodendrocytes and neurons in neurological disease principally by virtue of their agonist activity at *N*-methyl-d-aspartic acid receptors. The latest evidence is presented and discussed. The enzymes that control the checkpoints in the KP represent an attractive therapeutic target, and consequently several KP inhibitors are currently in clinical trials for other neurological diseases, and hence may make suitable candidates for MS patients. Underpinning these drug discovery endeavors, in recent years, several advances have been made in how KP metabolites are assayed in various biological fluids, and tremendous advancements have been made in how specimens are imaged to determine disease progression and involvement of various cell types and molecules in MS.

## Rationale for Involvement of KP in MS

Multiple sclerosis (MS) is a chronic, inflammatory demyelinating disorder of the central nervous system (CNS) whose etiology remains multifactorial and the subject of intense debate. This complexity arises from the several distinct demyelinating disorders of varying severity that are grouped under the general definition of MS. This indicates that definitive initial triggers (genetic, environmental, and others) that initiate episodes of autoimmune demyelination have yet to be identified, and that different mechanisms could contribute to lesion formation and tissue injury (1). MS is mediated by pathogenic T cells that are autoreactive against myelin antigens and coincides with broader neurodegenerative processes. Following trafficking into the brain *via* a compromised blood–brain barrier (BBB), T cells target and attack the myelin sheath of oligodendrocytes, the myelin-forming cells of the CNS, which envelop central neurons and axons (2). The inflammatory plaque is the pathological hallmark of MS and can be identified using magnetic resonance imaging (MRI) or histopathologically (3). MS represents one of the most common causes of chronic neurological disabilities in young people, and its course is greatly variable (4). MS is subclassified into at least four distinguishable categories based on the course of disease. Approximately 85% of MS patients have a disease course that is marked by episodes (relapses) of neurological symptoms followed by remission periods where symptoms recover or disappear. This relapse-remitting MS form (RRMS) is often followed by secondary progressive MS (SPMS), where the disease progresses to constant neurological deterioration with no period of remission. Primary progressive MS (PPMS) affects around 10% of patients who present with gradually increasing neurological disability from the onset. Similarly, progressive-relapsing MS (PRMS), which at 5% incidence is the rarest form, is also progressive, however, displays intermittent episodes of exacerbated symptoms. There are currently few drugs available to treat the progressive forms of disease (PPMS, SPMS, and PRMS), and therapies for RRMS have little efficacy in treating disability and neurodegeneration (5, 6).

**Figure 1:**
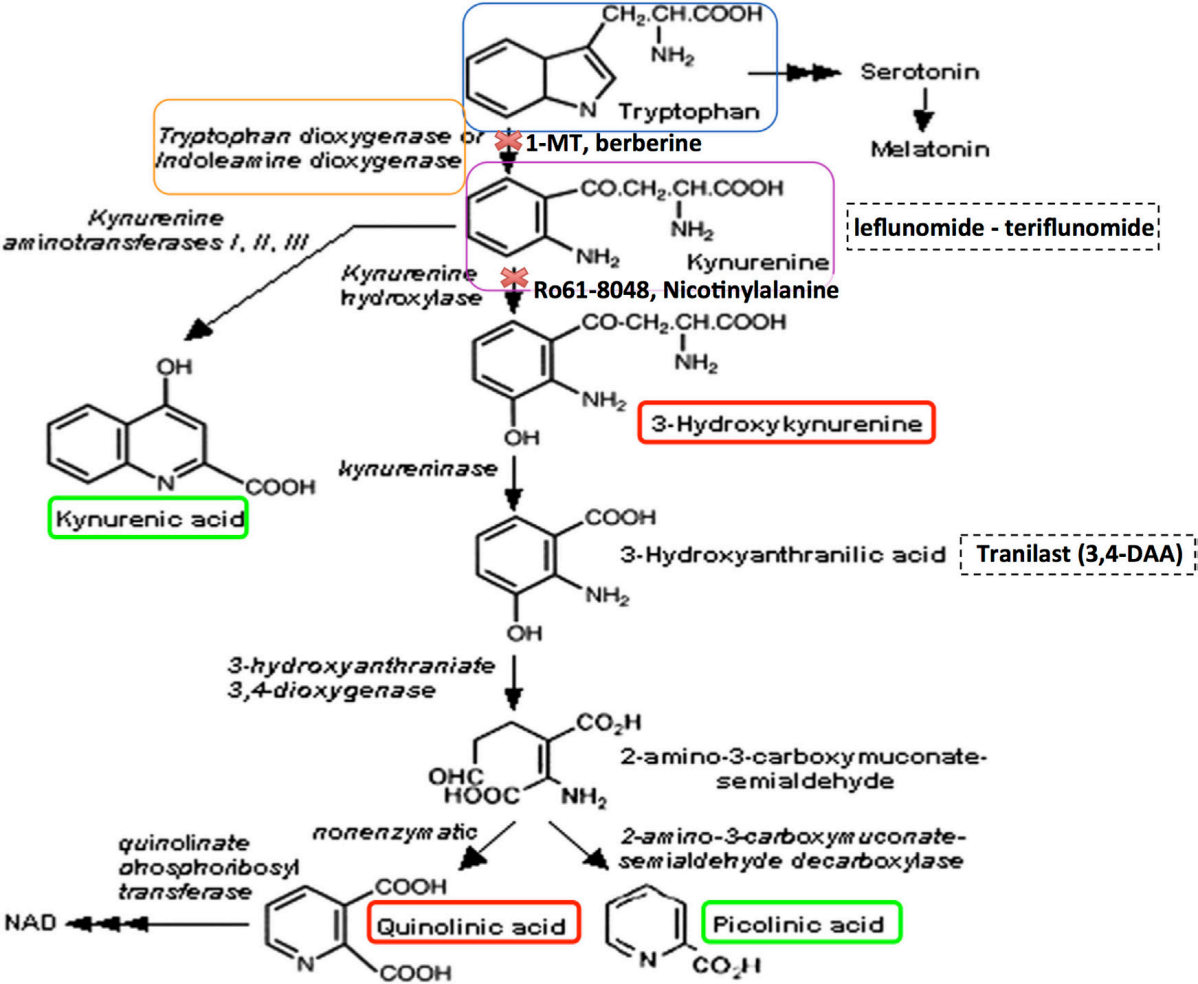
**The kynurenine pathway of tryptophan metabolism produces neuroprotective as well as neurotoxic metabolites that can influence MS pathology**. Neurotoxic metabolites are circled in red, and neuroprotective metabolites in green. Tryptophan (blue box) may be metabolized to serotonin and melatonin in multi-step sequential reactions, or alternatively is metabolized *via* the KP. This reaction is inhibited by 1-methyl tryptophan (1-MT) or berberine. Kynurenine (purple box) is the initial rate-limiting KP product of tryptophan metabolism by the enzymes indoleamine-2,3-dioxygenase (IDO-1) and tryptophan dioxygenase (orange box). Kynurenine is then converted *via* kynurenine aminotransferases (KATI/II/III) to kynurenic acid, a neuroprotective molecule as it antagonizes glutamate receptor-induced neurotoxicity. 3-hydroxykynurenine is produced by further metabolism of kynurenine, for which evidence is accumulating of its neurotoxic capability. This reaction is inhibited by Ro61-8048 or nicotinylalanine. Leflunomide (Avara^®^) is an immunosuppressive and anti-inflammatory drug. Teriflunomide is the active metabolite of leflunomide. These kynurenine analogs are effective in reducing active lesions in both rodent models and in a phase II clinical trial (235). Kynureninase catalyzes the conversion of 3-hydroxykynurenine to 3-hydroxyanthranilic acid. Tranilast is a synthetic anthracillic acid derivative drug with anti-inflammatory action (236). Sequential conversion to 2-amino-3-carboxymuconate-semialdehyde is the penultimate step leading to enzymatic production of (neuroprotective) picolinic acid, and the (non-enzymatic) production of the well-known neurotoxic compound quinolinic acid (QUIN). Further conversion of QUIN to the essential cofactor NAD^+^ is catalyzed by quinolinate phosphoribosyltransferase (QPRT). Dashed boxes indicate synthetic compounds, some of which are in drug development, that are derivatives of the KP metabolite described above.

Although an inflammatory aspect of the disease is clear – characterized by the presence of infiltrating macrophages and activated microglia around lesions (7, 8) as well as the autoimmune component arising from lymphocyte entry into the CNS, the role of other cells, such as monocytes, and other pathways that can further compromise oligodendrocyte health and contribute to the pathology of MS is increasingly being recognized by a theory of MS as a neurodegenerative disease with an autoimmune component (9). The question of whether inflammation leads to neurodegeneration or whether these are two different processes is currently unclear. The kynurenine pathway (KP) is activated in number of inflammatory and neurodegenerative diseases, including MS, and as such represents a common pathological mechanism highly relevant to our understanding of MS pathology (10). While the KP is the principal means by which tryptophan (TRP) is catabolized, it also leads to the production of several potent immunomodulatory and neuroactive intermediates, collectively called the kynurenines. Dysregulation of many of the enzymatic steps in the KP can favor the production of neurotoxic vs. neuroprotective metabolites (11). Monocytes, in particular, can be activated by high levels of inflammatory cytokines, which upregulate the expression of KP enzymes, favoring the production, and secretion of neurotoxic metabolites such as quinolinic acid (QUIN) (Figure 1, red box) (12).

Dysregulation of the KP may not be the primary cause of MS; rather the evidence thus far suggests its involvement is characterized by inflammatory episodes triggering KP activation (particularly in monocytes), trafficking to the brain, concomitant TRP degradation and production of neurotoxic metabolites. These aspects can contribute to the pathogenesis and the disease course of MS by promoting brain cell dysfunction and death, which in turn prevent the induction of essential brain cell survival and repair mechanisms. This is evidenced by studies showing that the KP is activated in early stages of MS in patients and in the experimental autoimmune encephalomyelitis (EAE) rodent model of MS (13), while monocytes are highly present in MS lesions during autoimmune episodes. Moreover, differences in disease course and clinical activity in MS are reflected by changes in the levels of KP metabolites, particularly in cerebrospinal fluid (CSF) (see CSF: A Window to Study Dysregulation of KP in the Pathology of MS and KP Metabolites Correlate with Increased MS Severity: Potential Utility as MS Biomarkers). Therefore, an activated KP could compromise the effectiveness of MS treatments, which predominantly target the autoimmune component.

In this review, we focus on evidence accumulated from MS studies that have demonstrated a dysregulation of the KP, resulting in elevated levels of neurotoxic metabolites both in the plasma and brain parenchyma (detected in CSF), and thus contribute to the progression of MS pathology. We focus on the cells that produce these damaging metabolites (Figure 1); circulating monocytes (see Peripheral Blood Monocytes as a Potent Source of Inflammatory KP Metabolites in MS) which enter the brain *via* a compromised BBB (see The BBB Performs a Critical Role in Maintaining Barrier Integrity and Permeability, Which Is Lost in MS) and to a lesser extent endogenous brain cells; how these metabolites are measured by analytical sampling (see CSF: A Window to Study Dysregulation of KP in the Pathology of MS) and also the mechanisms of action of these metabolites (see Mechanisms of Toxicity of KP Metabolites) and how they cause oligodendrocyte death (see Oligodendrocytes as a Target Cell for Elevated Levels of KP Metabolites in MS). We also consider how the KP can regulate both adaptive and innate immune responses (see KP Influence on the Immune System), and how some KP metabolites can serve as potential biomarkers of MS progression, detailed evidence for their association with MS (see KP Metabolites Correlate with Increased MS Severity: Potential Utility as MS Biomarkers) and therapeutic interventions (see Modulation of the KP as a Therapeutic Strategy in MS). Concurrent with this discussion, we also consider how advances in microscope imaging and allied techniques (see Microscope Imaging as a Central Tool for Advancing Knowledge of MS Pathology) have made a profound and ongoing contribution to our understanding of MS pathology.

## The Kynurenine Pathway

Approximately 95% of TRP is catabolized *via* the KP in both the CNS and periphery, which is thus the canonical route, while the remainder forms a substrate for serotonin and melatonin synthesis. In the KP, TRP is converted to *N*-formyl-l-kynurenine by indoleamine 2,3-dioxygenase (IDO-1/IDO-2) and tryptophan 2,3-dioxygenase 2 (TDO); the rate-limiting enzyme in TRP degradation. TDO is strongly and constitutively expressed in the liver; however, it is also expressed at lower levels in neurons, astrocytes, and endothelial cells (14, 15). Therefore, extra-hepatically, IDO-1 is the predominant enzyme in several different cell types, including monocytes, macrophages, microglia, astrocytes, neurons, and in some stem cells. IDO-2 is structurally and enzymatically similar to IDO-1; however, it is thought to function as a redundant enzyme to IDO-1 given its basal expression in a narrow range of cell types (16). Recently, new evidence suggests that IDO-2 has a role in “self-antigen” tolerance in autoimmunity and shaping immune tolerance in humans [reviewed in Ref. (17)]. Proceeding along the KP, *N*-formyl-l-kynurenine is metabolized by formamidase to l-kynurenine (KYN), the first stable intermediate metabolite (Figure 1). In the CNS, ~40% of KYN is locally produced, whereas 60% of KYN present is absorbed from the blood (18). Kynurenine is a central KP metabolite, capable of being degraded through three specific pathways to generate different metabolites [kynurenic acid (KYNA), 3-hydroxykynurenine (3-HK), and anthranilic acid (AA)]. Many of the kynurenines display neuroactive properties. In particular, the neurotoxic metabolites, the *N*-methyl-d-aspartic acid (NMDA) receptor agonist and excitotoxin, QUIN, the free radical generators, 3-hydroxykynurenine (3-HK), 3-hydroxyanthranilic acid (3-HA), and the neuroprotectants, picolinic acid (PIC), and KYNA, have significant associations with disease (19, 20), while the essential cofactor nicotinamide adenine dinucleotide (NAD^+^) is a very important end metabolite produced by catabolism of QUIN by the enzyme quinolinate phosphoribosyltransferase (QPRT).

## Triggering Agents of the KP in MS

Central nervous system inflammation and/or degeneration can trigger metabolism of TRP to produce kynurenine, and subsequent neurotoxic metabolites. Cells, such as monocytes and microglia, express all the KP components, whereas neurons express a restricted set and astrocytes lack expression of kynurenine 3-monoxygenase (KMO) resulting in high accumulation of kynurenine, a substrate for macrophages to further metabolize (12, 21–25). IDO-1 and TDO are the two enzymes that initiate TRP metabolism (Figure 1) and are regulated by different mechanisms. TDO is induced by corticosteroids and glucagon (26), whereas IDO-1 is induced by proinflammatory cytokines during an immune response. The inflammatory mediators that activate KP through IDO-1 induction include interferon (IFN)-γ (20, 27), interleukin (IL)-1, tumor necrosis factor (TNF)-α (28), cytotoxic T lymphocyte-associated antigen-4 (CTLA-4) immunoglobulin (29), toll-like receptor (TLR) (30) ligands polyinosinic:polycytidylic acid, lipopolysaccharide (LPS) (31), and unmethylated cytosine phosphatidyl guanosine (CpG) motifs (32). Although IFN-γ is regarded as the primary inducer of IDO-1, the regulatory mechanisms of IFN-γ mediated IDO-1 induction can be potentiated synergistically by other proinflammatory cytokines, such as IL-1, TNF-α, IL-1β, and TLR agonists, resulting in synergistic enhancement of IDO-1 expression (28, 33–36).

There is limited evidence that other enzymes within the KP can also be induced by proinflammatory cytokines, particularly by IFN-γ. Apart from the induction of IDO-1, IFN-γ is able to increase kynureninase (KYNU) activity in murine macrophages but not in microglial cells, which is of particular interest as TRP degradation by IDO-1 may not be the only enzymatic step controlling this pathway in activated macrophages (37). The enzyme diverting the KP toward the neurotoxic branch instead of KYNA production, KMO, is also increased through IFN-γ in activated macrophages (38) and in the brains of immune-activated macaques (39). Finally, in the human hippocampal progenitor cells, IL-1β treatment increased KMO and KYNU transcript levels (40). At millimolar concentrations, PIC acted as a macrophage coactivator by inducing macrophage inflammatory proteins 1-α and 1-β in conjunction with IFN-γ in the induction of reactive nitrogen intermediate production (41–43). The complex interaction between PIC and IFN-γ highlights the importance of its involvement in inflammatory response (41) in neurodegenerative conditions. Interestingly, the current disease-modifying agent of RRMS, IFN-β1b also induces KP metabolism in human macrophages and may be a limiting factor in its efficacy in the treatment for MS (21).

## CSF: A Window to Study Dysregulation of KP in the Pathology of MS

**Figure 2:**
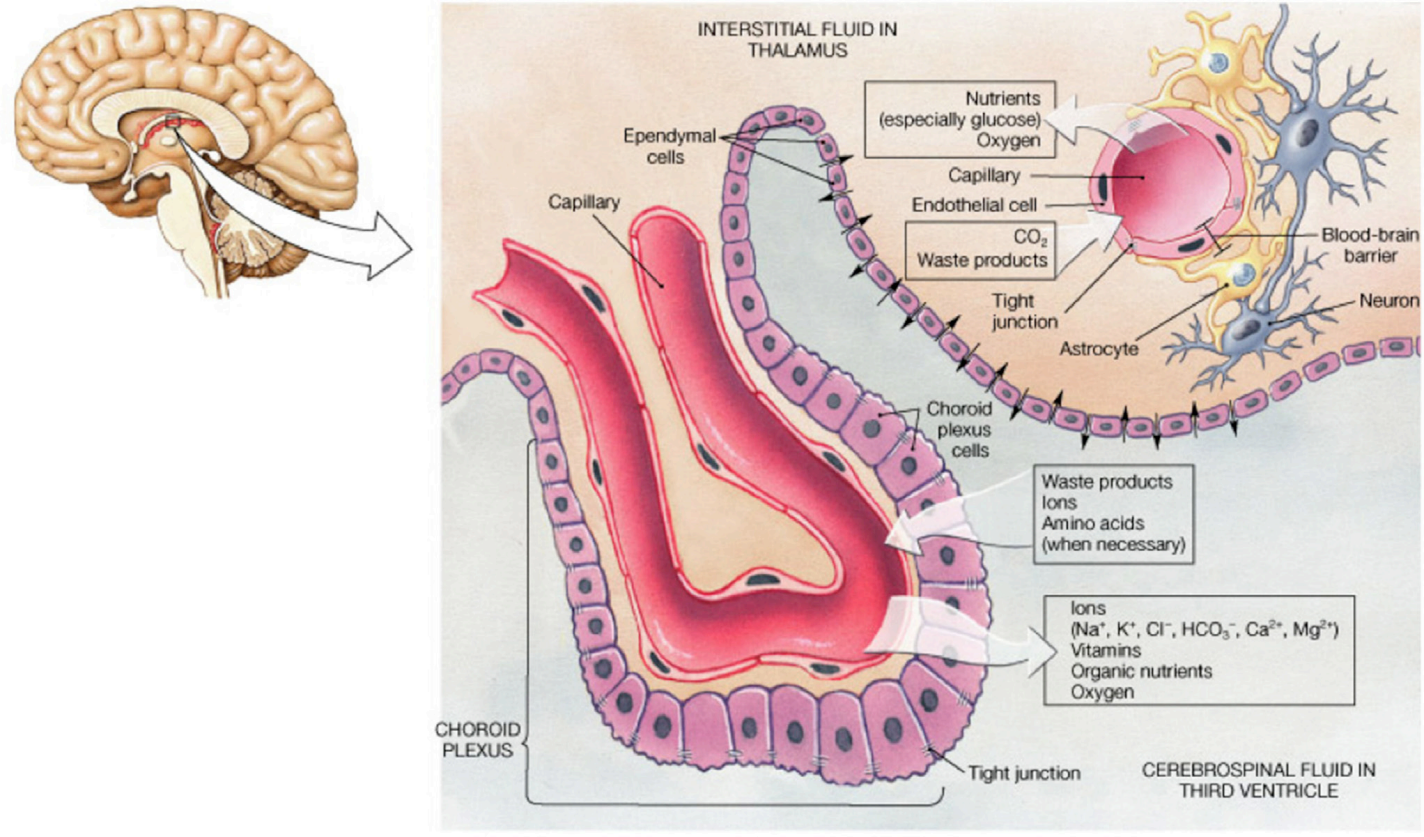
**Schematic summarizing the arrangement of cells within the choroid plexus and the physiology of exchange of solutes relevant to the understanding of how KP metabolites are found in CSF**. From website http://jonlieffmd.com/blog/the-very-intelligent-choroid-plexus-epithelial-cell

Cerebrospinal fluid bathes the inner ventricles of the cortical subventricular zone (SVZ) and subarachnoid space. CSF is a complex mixture of water, secreted proteins, enzymes, antibodies, peripheral blood, immune cells (e.g., B and T cell subsets), etc. and is constantly turned over, providing a sink for elimination of wastes from the interstitial fluid of the brain. This process is critical to proper brain homeostasis (44), and abnormal states are linked with multiple neurological diseases (45, 46). Choroid plexuses (47) are specialized structures located in the borders of the ventricles, facing the lateral wall, and consist of arrangements of epithelial cells (48), which secrete CSF and contain many villi which project into the ventricular space, vascular capillaries, neuronal contacts (49), and other supporting cells (Figure 2). Choroid plexuses, therefore, form a functional interface between the blood and CSF circulation and the bidirectional diffusion of important molecules, models of which are constantly evolving (50). Increasing evidence exists of alterations in choroid plexus function in various inflammatory CNS diseases (51).

From diagnostic, prognostic, and therapeutic aspects, CSF presents a unique opportunity to sample the content of fluid circulating around the brain and cerebrovascular interfaces (52–56). For many years, we have utilized CSF from patients of various neurological diseases to assay for the concentrations of KP metabolites as a discrete compartment separate from the blood plasma (for which concentrations of KP metabolites are not an accurate measure of brain levels). Our standard techniques developed and improved over the years include analysis by sensitive methods, such as high-pressure liquid chromatography (HPLC) and gas chromatography-mass spectrometry (GC-MS), to determine KP metabolite levels (12, 14, 20, 21, 24, 25, 57–62). While analyses using these methods are extremely sensitive and able to accurately quantify KP metabolites in microliter-sized aliquots, due to BBB breakdown in MS (detailed further in Section “The BBB Performs a Critical Role in Maintaining Barrier Integrity and Permeability, Which Is Lost in MS”), the obtained data reflect the heterogeneity of such samples by influence of these factors and thus the extent of the pathology, and should be considered in concert with other pathological information. Furthermore, our previous study of brain microvascular endothelial cells (BBB endothelial cells) and pericytes uncovered that these cells express components of the KP that vary depending on the presence of inflammatory stimuli such as IFN-γ or TNF-α. After stimulation, both BBB endothelial cells and pericytes produced KYN that could potentially act as a substrate for the production of damaging KP metabolites in other neighboring cells (63). Other biomarkers within CSF related to MS pathology are also being progressively developed which would not be considered further here, e.g., Ref. (56, 64–66).

### KP Metabolites Correlate with Increased MS Severity: Potential Utility as MS Biomarkers

In the EAE model of MS, significantly elevated levels of QUIN and the KYN/TRP ratio in rat serum were observed and these levels correlated with increasing disease severity (67). IDO-1 expression was demonstrated to play a role in remission of acute MS in the EAE model, suggesting that the KP enzymes and metabolites could be involved in regulating disease course in MS (68). In patient samples, the first report of the potential involvement of KP activation in MS pathology was several decades ago, when decreased levels of TRP were found in plasma and CSF of MS patients (69). This involvement was confirmed with numerous subsequent studies showing alterations in KP metabolites in RRMS patients. These include evidence that patients showed decreased levels of neuroprotective KYNA in the CSF during the remission phase but became elevated during remitting, acute phases compared with healthy controls (70–72). While KYNA levels in humans are highly varying in concentration (73), an upregulation of KAT enzyme expression as a neuroprotective mechanism could be a possible explanation for this, hence more studies are needed.

Given that KP activation is modulated by proinflammatory factors including IFN-γ, it is expected that MS patients in the acute phase (compared with the later chronic neurodegenerative phase) exhibit CNS inflammation with greater involvement of KP metabolites in disease progression (1). A comprehensive study into the relation between several KP metabolites and neurocognitive symptoms is only now beginning to unravel the subtle distinctions in the subtypes of MS. RRMS patients show higher levels of QUIN in the relapse phase vs. during remission (74), suggesting that QUIN is a potential biomarker of active demyelination phases. Indeed, RRMS patients in remission did not show KP metabolite levels different from controls, suggesting that kynurenine dysregulation is most prominent during symptomatic periods. Interestingly, PPMS showed increased concentrations of QUIN, TRP, and KYN, whereas SPMS had decreased levels of TRP and KYNA indicating that underlying pathogenic mechanisms that occur in PPMS may be distinct from those in SPMS. Notably, PPMS and the inflammatory control group (containing inflammatory disorders of the CNS) displayed similarities with amyotrophic lateral sclerosis, supporting the working hypothesis that alterations in KP metabolites are a common pathogenic mechanism across inflammatory diseases. Importantly, Hedegaard et al. has shown that MS patients sera, and not healthy controls, contain anti-myelin basic protein (MBP) autoantibodies that facilitate IFN-γ production (75). In line with this finding, a study examining changes in IDO-1 activity and expression in peripheral blood mononuclear cells (PBMCs) of RRMS patients found high IDO-1 expression and serum neopterin (a marker of inflammation) with a concomitant decrease in IFN-γ in the relapse phase of MS, but not in the stable, remitting phase of disease (76). This suggests that inflammation and KP activation are both mechanisms that are reflected in disease relapse and appearance of clinical signs. Current treatments that aim to slow the progression of MS, including IFN-β 1a and 1b and glucocorticoids, also alter KP metabolite levels. RRMS patients treated with IFN-β were found to have increased levels of neuroprotective KYN compared with untreated RRMS patients (77), whereas glucocorticoid treatment significantly reduced IFN-γ levels and IDO-1 expression.

## The BBB Performs a Critical Role in Maintaining Barrier Integrity and Permeability, Which is Lost in MS

The BBB and blood–cerebrospinal fluid barrier (BCB) are complex microvasculature barriers for the CNS and systemic circulation. These barriers provide protection, nutrient, and oxygen supply to the CNS. Under physiological conditions, the BBB (that surrounds parenchymal venules) and BCB (that surrounds the choroid plexus) protect the CNS from peripheral immune cell infiltration. The tight junctions (TJ) between the endothelial cells (of the BBB) and epithelial cells (of the BCB) restrict access of circulating cells to the CNS. Nevertheless, even in healthy brains, T cells can carry out immune surveillance of the CNS because they express adhesion molecules, chemokine receptors, and integrins that allow them to cross these barriers (78). On the other hand, the non-CNS targeted T cells are also capable of altering permeability and glial cell activity. Previous studies have shown that ovalbumin (OVA)-specific T cells are able to disrupt barrier integrity of the brain (79) and retina (80). Using an MRI approach, another group investigated *in vivo* T cell transmigration in relation to the BBB disruption on CNS tissues in a model using OVA- and proteolipid protein (PLP)-specific T cells, finding that antigen specificity (and not absolute number of infiltrating cells) is a critical determinant of the extent of BBB breakdown (81). Cerebral microvascular endothelial cells are also joined by TJ complexes with associated pericytes and astrocyte processes. During MS, damage of TJ proteins facilitates leukocyte infiltration, leading to oligodendrocyte death, axonal damage, demyelination, and lesion formation. Glial cell activation and further leukocyte invasion cause myelin damage and axonal degradation. Production of cytokines worsens BBB damage leading to progressive disability (82).

The CP (Figure 2) is a highly vascularized brain structure located within brain ventricles and consists of an epithelial layer forming a tight BCB, surrounding a core of fenestrated capillaries and connective tissues. The fenestrated capillaries are surrounded by CP epithelial cells, which confer tight barrier properties and restrict the entry of immune cells in the CSF. A local impairment of the endothelial cells and chemokine production results in a reduction in barrier integrity of the BCB, which are prominent events for early invasion of immune cells into the CSF. Indeed, the presence of oligoclonal bands from B cells in Western blots from CSF is seen in ~90% of MS patients and is a common test for confirmation of MS diagnosis (52). After passing the BCB, CSF-infiltrated leukocytes produce large amount of cytokines and activate endothelial cells of the brain vasculature, inducing expression of adhesion molecules and chemokines, leading to the formation of inflammatory lesions (83).

### BBB Breakdown and Entry of Immune Cells as a Key Hallmark of Early MS Pathology

A damaged BBB allows infiltration of autoreactive T cells and monocytes into the brain parenchyma. In MS, leukocyte infiltration into the CNS parenchyma is one of the earliest hallmarks (80, 84) and is thought to play a fundamental role in the development of the disease, including contributing to the early stages of lesion formation. BBB breakdown is also found in relapses (85–87); indeed, optic neuritis (ON) was the condition in which this was first noticed. Examination of the whole-mount retina preparation from EAE Lewis rats visualized the presence of BBB breakdown, cellular infiltration, and microglial activation as the earliest abnormal events in ON. This study also correlated the intensity of the immune response with the number of infiltrated leukocytes and microglial activation in the retinal parenchyma (84). Inflammatory cells are found to predominantly colocalize within the disrupted BBB (88, 89). Activated inflammatory cells (ED1^+^ monocytes, CD4^+^, and CD8^+^ T cells) in the lumen of affected vessels are capable of disrupting BBB permeability (84). Overall, these studies emphasize that abnormal BBB permeability and leukocyte infiltration in the CNS are key events leading to pathogenesis of MS. As discussed in greater detail in Section “Peripheral Blood Monocytes as a Potent Source of Inflammatory KP Metabolites in MS,” high expression of KP enzymes in activated monocytes, and their translocation into the brain parenchyma means they are poised to exert a profound impact on the survival of oligodendrocytes and neurons, and hasten the progression of lesion development and MS pathology.

Although these previous studies have provided ample evidence that BBB breakdown is the earliest event leading to MS pathology, these findings have been reinforced by modern MRI of the brain. For example, serial MRI studies have demonstrated that abnormalities of the BBB may precede myelin damage and leukocyte infiltration (90). Histopathological (91–94) and serial MRI (90, 95) studies indicated that structural changes may precede myelin damage and leukocyte infiltration. Cramer and colleagues use a sensitive dynamic contrast-enhanced (DCE) MRI concluded the importance of a BBB defect in MS. MS lesions are predominantly located in the periventricular normal appearing white matter (96, 97). The presence of ON and lesions can be predictive of MS progression, as an MRI-based risk stratification showed that patients with MRI T2 lesions and ON progressing faster to full MS compared with patients with ON alone (56 vs. 22%) (98).

Subsequently, Cramer and colleagues showed that the BBB permeability is able to predict conversion from ON to MS within 2 years, in a group of patients presenting with monosymptomatic ON- and T2-lesion count compared with another group presented with T2-lesion count alone (99). Subtle disruption to the BBB is often found at discrete locations in the brain, for which modern radiographical imaging modalities such as MRI can be utilized (100). Kawakami and Flugel also used intravital two-photon imaging to measure BBB permeability. Using this imaging technique, they examined infiltration of autoreactive T cells across the intraluminal surface of CNS blood vessels in animal model of MS (101).

## KP Influence on the Immune System

One of the most profound roles of the KP has been its implication in the pathological regulation of both the innate and adaptive the immune system (18, 102). IDO-1 is considered the major contributor to the immunoregulatory functions of the KP due to the depletion of TRP and the production of kynurenine metabolites. IDO-1 is expressed in several types of immune cells, including microglia, monocytes, and macrophages, and can be readily induced by interferons, most effectively by IFN-γ (102). IDO-1 activation has potent antimicrobial effects, which occurs partly through the depletion of the essential amino acid TRP. IDO-1 plays a vital function in maintaining polymorphonuclear cells effector function against pathogens (103). Conversely, IDO-1 can also suppress the immune response leading to immunological tolerance, which mediates various phenomena such as allograft acceptance, tumor camouflage, and maternofetal tolerance (18).

To date, at least three mechanisms that initiate immunological suppression are known. These immunosuppressive effects all correspond to IDO-1 activation and its downstream effects in certain populations of T cells. First, TRP levels are depleted following IDO-1 induction, which inhibits the proliferation of reactive T lymphocytes and increases their susceptibility to apoptosis (104). Second, the resulting increase in kynurenine metabolites (KYN, QUIN, and 3-HAA) interferes with proliferation and initiates selective apoptosis of T helper 1 (T_H_1) lymphocytes, which are responders to antigen-presenting cells (105, 106). Notably, there is a preferential inhibition for T_H_1 cells by IDO-1 activation, although the activation of regulatory T-cells may also impede T_H_2 cells (102). There are controversial data surrounding QUIN’s effect on T cell regulation, although it is currently thought that this process relies on a TRP-deficient microenvironment (105, 107, 108). KYN has been found to moderately impair the killing ability of natural killer cells, whereas KYN and 3-HAA both exert proapoptotic and suppressive effect on these cells (105, 106, 109). Additionally, TRP depletion and kynurenine metabolites act synergistically to downregulate expression of the T cell receptor ζ-chain on CD8^+^ T lymphocytes consequently reducing their cytotoxic capabilities (110). Third, the combination of the presence of kynurenine metabolites and TRP depletion increases the number of regulatory T cells positive for forkhead box P3 (FOXP3^+^) *via* TGFβ induction and its impact on naive T cells (110). Moreover, downstream TRP catabolites are able to shift dendritic cells to a tolerogenic phenotype independent of the microenvironment TRP levels, i.e., without functional IDO-1 (111, 112). Therefore, IDO-1 competent dendritic cells also contribute to KP-mediated immune-suppression by contributing to a tolerogenic environment. Together, this plays a substantial role in the development of immune tolerance and the induction of a negative feedback loop that regulates the immune response (113).

### KP and Immune Modulation in the Pathomechanism of MS

The importance of IDO-1 in immune modulation particularly to counteract autoimmunity has been illustrated in the EAE mouse model. Autoreactive CD4^+^ T_H_1 cells and T_H_17 cells mediate the autoimmune characteristics present in CNS inflammation in MS and in the EAE animal model (114). Indeed, there is widespread evidence that shows the potential of IDO-1 activation to reduce autoimmune inflammation in the CNS. Furthermore, pharmacological inhibition or genetic ablation of IDO-1 exacerbates EAE clinical scores, associated with decreased T_Reg_ cell responses and increased T_H_1 and T_H_17 responses (68, 115, 116). Conversely, the clinical symptoms of EAE can be ameliorated by administration of 3-HAA or its synthetic derivative, Tranilast, and are likely associated with an enhanced expression of TGFβ by 3-HAA. This possibly leads to an increase in the number of T_Reg_ cells, which can suppress the responses of autoreactive T cells, including the T_H_17 response (115). Furthermore, Xiao et al. reported that dendritic cells pretreated with IFN-γ alleviated the histopathological and clinical characteristics of EAE (117). It is hypothesized that IDO-1 activation is a self-limiting mechanism as both IDO-1 and KMO are induced by the autoreactive, IFN-γ secreting T_H_1 cells (37). This response could be designed to counteract the detrimental effects to the pathological elevation of kynurenine metabolites. This is supported by the observation of toxic levels of QUIN and 3-HK that are reached in the spinal cord but not the brain in EAE animals with concomitant increased activity and expression of KMO (67, 117, 118).

The neuroinflammatory process can be significantly decreased by inhibition of IDO-1 enzyme activity, considerably reducing disease exacerbation (119). Monaco et al. revealed corresponding clinical evidence of depressed TRP levels in both serum and CSF of MS patients (69). A subsequent study has yielded conflicting results to these findings, however, a negative correlation between neopterin, a marker of macrophage activity, and TRP levels in the CSF was observed, reflecting IFN-γ-induced macrophage induction and IDO-1 activation, respectively (120). Although an additional clinical study this year found an increase in IDO-1 activation *via* QUIN/KYN ratios in MS patients compared with control, there were no significant differences in kynurenine metabolite levels between MS and other neuroinflammatory disorders. However, some patterns emerged upon stratification of disease into acute and chronic phases of disease course such as a high QUIN/KYN ratio in RRMS patients who are in relapse compared with the remission phase. This suggests a high degree of variation in the course of neuroinflammation in MS (74). Initial IDO-1 activation is thought to be beneficial in MS; however, there is an emergence of a double-edged sword that is present following prolonged exposure. During CNS inflammation, increased IDO-1 activity generates kynurenine metabolites that are neuroactive, specifically neurotoxic QUIN (121). Therefore, although IDO-1 activation acts as an anti-inflammatory, its induction could contribute to the neurodegenerative features in MS in the long-term.

### Peripheral Blood Monocytes as a Potent Source of Inflammatory KP Metabolites in MS

Current evidence suggests that excessive activation of the KP in mononuclear phagocytes can participate in the pathogenesis of MS. Activated monocytes and monocyte-derived macrophages (MDMs) are abundantly present in the demyelinating plaques of MS patients and their migration from the periphery to the CNS is necessary for the development of the MS mouse model, EAE. Mononuclear phagocytes, in particular circulating monocytes and MDMs, display high levels of pivotal KP enzymes and can up regulate their expression in response to inflammation (23). Monocytes display considerable plasticity, being found in various tissues and organs as resident macrophages, and patrolling forms with decreased expression of classical inflammation molecules (e.g., Ly6C in mice and CD14/16 in humans). Patrolling forms carry out immune surveillance of the endothelium and rarely extravasate into tissues without immune stimulus or tissue damage (122), in which case they are then alternatively regulated in classical inflammatory form [reviewed in Ref. (123)]. This makes them a potent source of neurotoxic KP metabolites (e.g., QUIN and 3-hydroxykynurenine) to contribute to MS pathology upon their migration and entry to the brain. Indeed, IFN-γ also caused upregulation of KMO and QPRT, two enzymes involved in the production of neurotoxic metabolites such as 3-HK and QUIN (12, 23).

Studies of the EAE model have reported a distinct rise in the level of Ly6C^high^ proinflammatory monocytes within the blood stream before onset of clinical signs. At disease onset, or during relapses, proinflammatory monocytes migrate to the CNS, where their numbers directly correlate with the severity of EAE symptoms (124–126) and in turn, with elevated levels of neurotoxic QUIN in the spinal cord (67). This migration might be facilitated by a damaged BBB, one of the earliest clinical findings in MS, thought to play a fundamental role in the development of the disease [see BBB Breakdown and Entry of Immune Cells as a Key Hallmark of Early MS Pathology; Ref. (86, 87)]. To confirm that monocyte migration to the CNS is involved in EAE pathogenesis, Mishra and colleagues have demonstrated that its inhibition by the candidate MS drug Laquinimod prevented the onset of EAE and its clinical signs (126). More recently, depletion of phagocytotic monocytes by clodronate treatment reduced the severity of EAE symptoms and protected against further axonal loss (127).

In humans, early postmortem studies of MS plaques have correlated the number of macrophages present in chronic demyelinating lesions to the severity of axonal damage responsible for the symptoms of the disease (128, 129). Indeed, monocytes have been found in acute demyelinating lesions and in the demyelinating edges of chronic lesions (130). Moreover, advanced microscopy, through 3D reconstruction of serial block-face scanning electron microscopy images, has been used in a pivotal study of EAE mice where Yamasaki and colleagues demonstrated that MDMs are directly in contact with the axoglial unit and begin demyelination, as opposed to microglia-derived macrophages that were only found adjacent to the lesions and participated in debris clearance (131).

**Figure 3:**
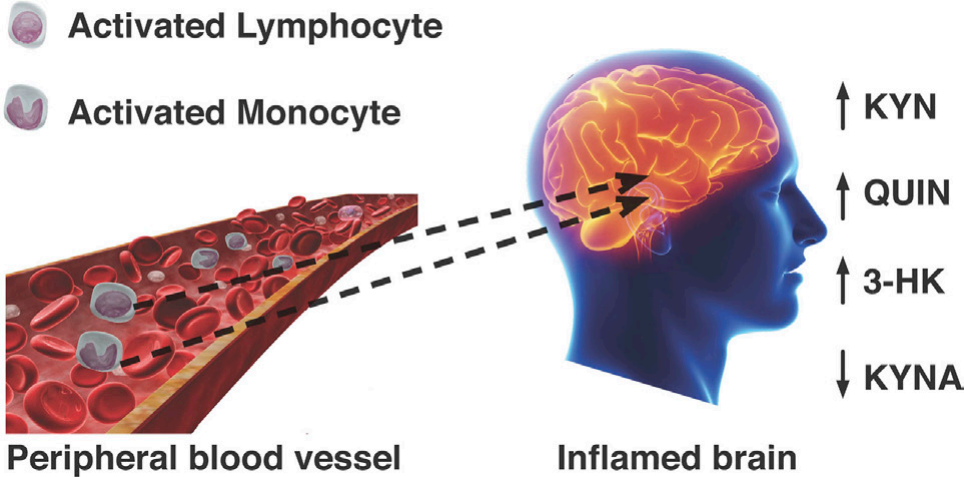
**Passage of activated lymphocytes and monocytes across a compromised blood–brain barrier leads to dysregulation KP metabolism**. Neurotoxic metabolites QUIN and 3-hydroxykynurenine are increased, while formation of neuroprotective kynurenic acid (KYNA) is not favored.

The importance of this study and of the evidence linking activated mononuclear phagocytes to MS is highlighted by different lines of evidence about the KP in these cells: first, the activated MDMs produce 19 times more QUIN than activated microglia (23); second, the CNS-resident cells do not possess the full enzymatic machinery of the KP and are unable to synthesize high quantities of QUIN, but can still produce KYN (14, 22, 62); third, extra KYN released by astrocytes in the brain can be used by monocytes and MDMs that traffic into the brain to synthesize more QUIN (22); fourth, the level of QUIN produced by monocytes and MDMs is toxic to neurons and to oligodendrocytes (20, 132). Overall, this evidence suggests that monocytes and macrophages migrated to the CNS and activated by IFN-γ (133) can act as a major reservoir for the secretion and accumulation of damaging QUIN in the brain and spinal cord of MS patients. Therefore, this lends support to the possibility that monocytes and MDMs participate in the pathogenesis of MS *via* dysregulation of the KP and excessive accumulation of QUIN and associated neurotoxicity of oligodendrocytes and neurons (summarized in Figure 3).

## Mechanisms of Toxicity of KP Metabolites

Several decades ago, it was suggested that the kynurenines could act as important endogenous modulators and that they may be involved in the pathogenesis of not only MS but also a number of neurodegenerative diseases such as Alzheimer’s, psychological disorders such as schizophrenia and depression, and neuroinflammatory diseases such as HIV-associated neurological disorder (HAND) (57, 59–61, 134–137). Three kynurenines in particular, QUIN, 3-HK, and 3-HAA, were noted for their ability to cause *in vitro* and *in vivo* neuronal death at slightly elevated concentrations. At low concentrations (≈50 nm), QUIN serves as a substrate for NAD^+^ production in neurons and astrocytes (132).

In pathological conditions, the concentrations of QUIN and 3-HK found in the CNS are significantly lower than the levels required to perturb neuronal survival (102). Rather, it is considered that these molecules become markedly potent neurotoxins during *chronic* exposure to low levels and those different populations of neurons are selectively affected by each agent (60). Chiarugi et al. demonstrated that in murine mixed cortical cells, prolonged exposure to QUIN and 3-HK from 24 to 72 h, significantly decreased neurotoxic thresholds from 100 to 1 μM, respectively, and that exposure to a combination of the two compounds also increased the neurotoxic effects (138). This is of relevance to pathological conditions where both QUIN and 3-HK are simultaneously released and concomitantly accumulate to levels that are neurotoxic leading to chronic exposure of CNS cells (7). QUIN neurotoxicity is primarily attributed to activation of the NMDA receptor and free radical production, and therefore shows complex patterns of neurodegeneration, while 3-HK and 3-HAA are accepted to have a primary role as pro-oxidant metabolites (Table 1) (138).

**Table 1:** **The mechanisms of toxicity following pathological increases in neurotoxic KP metabolites**.

Metabolite	Mechanism	Pathology
3-HK	ROS formation	Oxidative stress, apoptosis, potentiation of excitotoxicity
QUIN	Generation of free radicals, NMDA receptor activation	Excitotoxicity, free radical formation, mitochondrial dysfunction, apoptosis or necrosis, cytoskeletal destabilization
3-HAA	Generation of free radicals	Oxidative stress, apoptosis

### Quinolinic Acid

Under normal conditions, QUIN is present in nanomolar concentrations in the brain and is catabolized for the synthesis of NAD^+^. At low nanomolar physiological concentrations, QUIN is not toxic to neural cells; however, at elevated levels of QUIN (300 nM and possibly even as low as 100 nM with chronic exposure) that are found in inflammatory microenvironments, QUIN begins to be toxic (139). The pathological mechanisms of QUIN neurotoxicity have therefore been found in numerous neurodegenerative processes associated with neuroinflammation such as MS.

Two major factors that render QUIN a potent neurotoxin is the saturation limit of QPRT, and QUINs ability to act as an endogenous weak agonist on the NMDA glutamate receptor. The 3-HAO enzyme, which produces QUIN, has an 80-fold higher reaction velocity than QPRT, the enzyme which degrades QUIN (140). Furthermore, neuronal QPRT is saturated at QUIN concentrations that exceed 500 nM (141). This leads to the production of QUIN at a faster rate than its conversion to NAD^+^, causing the accumulation of toxic QUIN- and NMDA-mediated excitotoxicity (142). Furthermore, surrounding cells (astrocytes, neurons, and microglia) can take up excess released QUIN from the microenvironment, further promoting cellular damage. Moreover, astrocytes favor KYNA synthesis as they do not express KMO (25), whereas microglia preferentially form metabolites of the QUIN branch due to their low expression of KAT (102). Thus, astrocytes appear to maximize the synthesis of KYNA and alone are neuroprotective. However, it should be mentioned that it takes a threefold higher concentration of KA to antagonize the same amount of QUIN (143).

**Figure 4:**
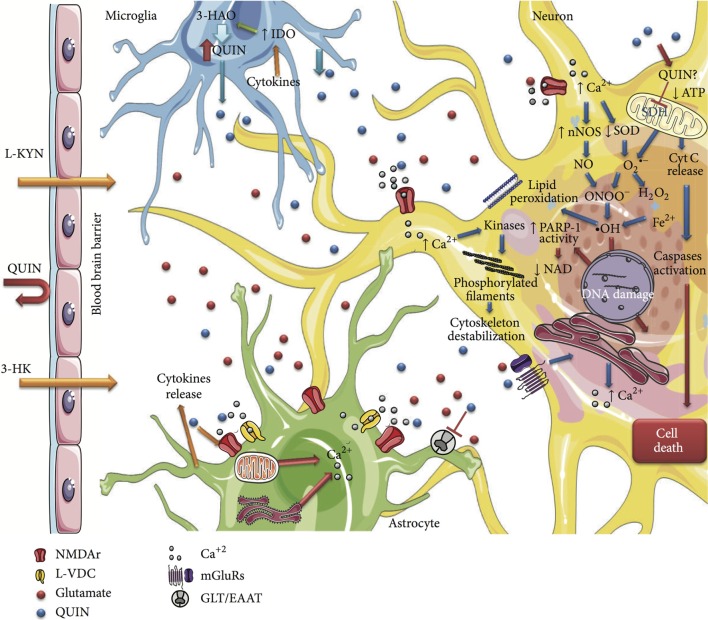
**Schematic summarizing the cytotoxicity mechanisms of QUIN in neural cells**. This figure is taken from Ref. (149), under Creative Commons license. The significant effects of QUIN on oligodendrocytes have been summarized separately (see Oligodendrocytes Express Only a Subset of KP Enzymes and Oligodendrocytes Are Particularly Sensitive to Quinolinic Acid Toxicity).

The low levels of QUIN in astrocytes are rapidly degraded. Indeed, the expression of QPRT by astrocytes is IFN-γ inducible and explains the rapid catabolism of QUIN in these cells (22). However, the presence of microglia or infiltrating macrophages, such as in MS, means that the high levels of KYN produced by astrocytes can be metabolized to QUIN by neighboring cells (Figure 4) (144). Low levels of neuroprotective PIC may also be synthesized in astrocytes, but production is severely compromised by IFN-γ stimulation (25).

### NMDA and Excitotoxicity

Excitotoxicity is a pathological process that results in neuronal damage and death caused by the overactivation of excitatory amino acid receptors. Excitatory amino acids are the primary excitatory neurotransmitters in the hippocampus and cerebral cortex and thus play crucial roles in the psychological functions of neurons. Neuronal excitotoxicity typically refers to the excessive exposure to glutamate, the major excitatory neurotransmitter in the CNS of mammals (145). QUIN is a selective agonist of NMDA receptors, specifically receptor subtypes are composed of NR2A and NR2B subunits. Therefore, as the hippocampus and striatum contain the widest distribution of NMDA receptors, they are areas of the brain most susceptible to QUIN neurotoxicity. Interestingly, neural stem cells (NSCs) in the adult human brain are localized to the subventricular zone of the striatum and the subgranular zone of the hippocampus (146). Considering the importance of adult NSCs in migration and maturation into oligodendrocytes following demyelination in MS, their location in the regions of the brain that exhibit the highest levels of QUIN neurotoxicity, suggests a relationship between KP overactivation and the inhibition of remyelination in MS. In support of this concept, Croitoru-Lamoury et al. found that IFN-γ stimulation and concomitant KP activation in mesenchymal stem cells (MSCs) diminished their proliferation and altered their capacity to differentiate. Similar to MSCs, NSCs also express the complete and functional KP enzyme machinery (147). In addition, there is a plethora of studies that have demonstrated that QUIN, as well as KYN and 3-HA, have significant effects on proliferation and activation in specific T cell subsets (105, 107, 148). These studies indicate a key role of the KP in controlling proliferation and differentiation in various cell types. If pathophysiological concentrations of QUIN do affect NSC proliferation and differentiation, it could provide a relevant mechanism by which remyelination is hindered in MS. Due to the rapid saturation of QPRT, the uptake of QUIN at the synaptic cleft can be delayed, causing further stimulation of the NMDA and continual damage (149). Prolonged activation of the NMDA receptors impairs calcium homeostasis, generates free radicals through the activation of nitric oxide synthesis, and leads to mitochondrial damage and initiates programed cell death (150). This induced death has been observed at pathological concentrations *in vitro* with rat oligodendrocytes (1 mM), primary human neurons and astrocytes (150 nM), and recently in motor neurons (100 nM) (132, 136, 151, 152). There is additional evidence showing that QUIN can induce NOS activity in both neurons and astrocytes leading to increases in both poly(ADP-ribose) polymerase (PARP) activity, extracellular lactate dehydrogenase (LDH) activity, and oxidative stress through an increase in production of free radicals (149).

Quinolinic acid toxicity is also due to its direct effect upon the glutamatergic system, which potentiates its primary mechanism of excitotoxicity. In hippocampal slices and cultured astrocytes, QUIN has been shown to increase glutamate release in the synapses, inhibit its re-uptake, and reduce glutamate to glutamine recycling by inhibiting glutamine synthetase activity. This elevates synaptic glutamate concentration and potentiates excitotoxicity by further overstimulating the NMDA receptor (153–157). Therefore, chronic QUIN exposure is similar to the pathological effects seen in the neurodegeneration of MS. This is in agreement with the findings of Flanagan et al., who found a causal relationship between the degree of clinical severity in the EAE model and the levels of QUIN in the spinal cord (67).

### Free Radical Production

**Figure 5:**
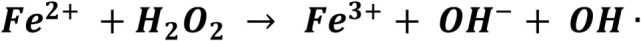
**The Fenton reaction for the production of reactive oxygen species**.

*N*-methyl-d-aspartic acid receptor activation does not account for all of the neurotoxic effects mediated by QUIN. Indeed, oxidative stress and the generation of free radicals can occur through NMDA receptor-dependent activation or independently by the formation of QUIN–iron complexes. It has been shown that the QUIN–Fe^2+^ complex mediates the formation of reactive oxygen species (ROS) through the Fenton reaction (Figure 5) leading to downstream lipid peroxidation and *in vitro* DNA damage (158).

This effect was attenuated when an alternative ligand for iron, the nucleoside analog acyclovir, was added. The removal of iron from QUIN inhibited lipid peroxidation and decreased the production of superoxide anion radicals demonstrating that the ligand identity is important to ROS development (159). Supporting this hypothesis, QUIN has also been shown to dysregulate redox homeostasis by affecting the endogenous antioxidants such as reduced glutathione, and depleting enzymes that scavenge free radicals, such as copper/zinc–superoxide dismutase (CuZn–SOD). QUIN has been shown to modify the activities of several endogenous antioxidants and deplete the activity of cytosolic CuZn–SOD, therefore exerting stress on primary antioxidant defense mechanisms (160). Moreover, these pro-oxidant effects of QUIN can be prevented by treatment with different antioxidants such as melatonin and pyruvate. The pro-oxidant toxicity of QUIN is likely to occur by decreasing the nuclear translocation of the transcription factor NF-E2-related factor 2 (Nrf2), and important transcriptional activator of antioxidant response element (ARE). The Nrf2/ARE pathway induces phase II antioxidant enzymes and is therefore an important promoter to detoxify oxidants. Tert-butylhydroquinone (tBHQ) exhibits antioxidant properties through its ability to induce Nrf2 nuclear translocation, thus activating ARE. In rat striatal slices, QUIN was observed to decrease nuclear Nrf2, while tBHQ protected against QUIN-induced mitochondrial dysfunction and lipid peroxidation, and partially recovered glutathione-*S*-transferase activity (161). This suggests that QUIN toxicity is also associated with a silencing of phase II antioxidant enzymes, thereby generating oxidative stress and simultaneously reducing antioxidant defenses.

In recent years, evidence implicates the roles of nitrative and oxidative damage and mitochondrial dysfunction in directly causing acute axonal damage in new inflammatory lesions in MS that may lead to degeneration (4, 162). An *in vivo* study using confocal microscopy of early lesions in the EAE found free radicals caused early mitochondrial damage at inflammation sites prior to demyelination (163). Of interest, scavengers of reactive oxygen and nitrogen species could reverse this injury, indicating a potential neuroprotective strategy. It is thought that oxidative and nitrative damage advances both the initial and chronic active lesion in MS. Indeed, Haider et al. found that in active MS plaques and not in control brain tissue, there were high levels of oxidized lipids and DNA. DNA oxidation occurred mainly in oligodendrocyte nuclei, which also exhibited signs of apoptosis. Additionally, DNA and lipid oxidation correlated significantly with inflammation, determined by quantifying human leukocyte antigen-D expressing macrophages and microglia and CD3^+^ T cells in the lesions (164). Given the key role of oxidative damage in driving MS pathology, and QUIN-mediated free radical production, this could constitute an additional means by which the KP contributes to MS.

However, more recent reports have suggested a dual role of QUIN in being able to both scavenge and produce ROS, depending on the chemical environment and its concentration (165). Other small molecules that act as antioxidants, such as specific vitamins and metabolites, also typically show this double behavior and likely play a relevant role in maintaining redox homeostasis and oxidative balance. In support of this, studies have indicated that lower concentrations of QUIN affect the redox homeostasis of iron maintaining the Fe(II)/Fe(III) equilibrium (166). Indeed, it has been suggested that at low QUIN concentrations, QUIN participates as an antioxidant and that the combination of high levels of ROS and QUIN are required for oxidative stress and cytotoxicity (166). It should be noted that these experiments examined QUIN activity in non-cellular based assays. The complex chemistry of QUIN will be largely milieu dependent and based on specific cellular environments.

### Cytoskeleton

Quinolinic acid has also been shown to induce damage to dendrites and axons with recent evidence showing that toxic QUIN levels phosphorylate structural proteins, thereby destabilizing the cytoskeleton (167, 168). The cytoskeleton is vital for neuronal cell shape and function and is involved in maintaining synapse formation, internal transport of molecules, and neurite outgrowth. Furthermore, it has been well established that axonal injury, including axonal transport disruption, is prevalent in active MS lesions (169). Acute intrastriatal administration of QUIN was found to cause NMDA-mediated Ca^2+^ influx and oxidative stress that resulted in the hyperphosphorylation of intermediate filaments in neural striatal cells (149, 168). In rat striatal slices, 100 μM QUIN altered the cytoskeletal homeostasis of both astrocytes and neurons. In astrocytes, QUIN’s actions were mediated by a rise in Ca^2+^ influx through L-type voltage-dependent Ca^2+^ channels (L-VDCC) and NMDA receptors, whereas in neurons, additional actions involved intracellular Ca^2+^ and metabotropic glutamate receptors. Both cases similarly result in a cascade of second messenger-dependent kinase activation, the phosphorylation of domain sites on neurofilament subunits and GFAP and irregular assembly of intermediate filaments in both glia and neuronal cells (167, 170, 171).

Additionally, work performed by Rahman et al. demonstrated that prolonged exposure to QUIN-induced significant changes to the structure of human neurons including decreasing organelles, dendritic beading, and the disruption of microtubules. The observed structural perturbations were associated with a decrease in major tau phosphatases expression and activity and consequently a concomitant increase in tau phosphorylation at multiple sites (168). In line with this evidence, Anderson et al. identified irregular tau phosphorylation in the EAE model and in progressive MS patients (169). Abnormal tau phosphorylation and insoluble tau accumulation is associated with both axonal and neuronal loss, which parallels the transition of relapse-remitting to the chronic, secondary progressive stage in EAE. Analysis of secondary progressive brain tissue in humans with MS revealed significant abnormal phosphorylated tau and insoluble tau formation. This observation was focused on areas dominated by demyelination, gliosis, and neuronal injury. Given that QUIN has direct effects on gliosis, neuron survival, and tau phosphorylation, this further supports a role for QUIN in the neurodegeneration associated with MS. Interestingly, QUIN may also have an effect on intracellular Ca^2+^ signaling as QUIN-induced NMDA receptor overstimulation causes early damage to the sarco/endoplasmic reticulum Ca^2+^-ATPase (SERCA) pump, thereby disturbing intracellular Ca^2+^ regulations (172).

### 3-Hydroxykynurenine

Similar to QUIN, the plasma, brain, and spinal cord levels of 3-HK are elevated in EAE rats (118). There is evidence that 3-HK is a neurotoxic metabolite and it, therefore, may have an important role in the neurodegeneration of MS (173). To date, there is a multitude of literature providing evidence that 3-HK is a pro-oxidant and a potent generator of reactive species that induces apoptosis. This characterization has been performed *in vitro* at concentrations that are supraphysiological, ranging from 10 μM to 1 mM. These concentrations are considerably higher than both normal brain (~0.08–0.3 μM) and pathological brain (0.3–1.2 μM) concentrations and could evoke toxicity not observed under normal conditions (113). In support of this, there is growing evidence suggesting that 3-HK is an endogenous antioxidant. Despite these considerations, 3-HK is described as neurotoxic and the dual effects of this metabolite will be discussed below.

In the presence of oxygen and at neutral pH, 3-HK easily undergoes auto-oxidation forming *o*-semiaminoquinone. The oxidation of 3-HK has been found to (1) generate ROS, which promotes lipid oxidation, protein modification, inflammatory response modulation, and DNA damage; (2) reduce trace transition metals, including Fe^3+^ and Cu^2+^, to pro-oxidants, Fe^2+^ and Cu^+^, capable of generating further radical formation in Fenton-like reactions; (3) *o*-semiaminoquinone readily reacts with oxygen generating quinone-imine, another highly reactive product which also participates in additional oxidative reactions (113). In line with this, 3-HK and 3-HAA (10 μM) induced neuronal cell death with apoptotic features following generation of ROS in primary striatal neurons. This toxicity was dependent on its cellular uptake by large neutral amino acid transporters in a sodium-dependent process and the increase in intracellular ROS, as various antioxidants inhibited this process (174, 175). Cell death occurred *via* p38 death signaling and was independent of caspase-3 mechanisms. However, cerebellar granule neurons appeared more resistant than striatal cells to HK-induced damage, suggesting toxicity specificity (250 μM) (176, 177).

Interestingly, when human astrocytes are treated with 3-HK at concentrations lower than 100 nM, intracellular NAD^+^ levels are significantly augmented. At doses above 100 nM, however, NAD^+^ levels are significantly decreased and extracellular LDH activity is increased (178). NAD^+^ is a molecule involved in many metabolic processes and is a vital cofactor for several enzymes. For example, NAD^+^ is a precursor for agents that mobilize calcium and regulate gene transcription through chromatin-associated protein modification and is a substrate for ADP ribosylation of proteins (179, 180). Therefore, alterations in 3-HK concentrations may indirectly change (1) gene expression, (2) DNA repair, and (3) intracellular Ca^2+^ levels.

There have been few studies of the *in vivo* effect of 3-HK. Of these, the most notable examined the synergistic possibility of the combination of 3-HK and QUIN in neurotoxicity. In rat brains, intrastriatial injection of 3-HK (5 nM) or QUIN (15 nM) individually caused no or marginal damage, whereas coinjection of the two metabolites caused impaired rotational behavior and significant increases in the volume of lesions (113). Notably, there was an absence in *de novo* generation of QUIN suggesting that the *in vivo* conversion of QUIN from 3-HK was not the mechanism behind the potentiation of QUIN toxicity (181). This could be a result of coinjection, as there is a possibility of chemical interactions that could modify the reactive components of each reagent (113). These findings might also indicate that the normal brains capacity to scavenge radicals is sufficient to counteract 3-HK-induced radical formation and prevent cellular apoptosis (182).

### 3-HAA

At present, there have been several studies that have examined 3-HAA activity in conjunction with 3-HK. It has been recognized that 3-HAA exhibits similar characteristics to 3-HK to generate superoxide anions by undergoing auto-oxidation and, thus, initiate apoptosis (183). Recent evidence has also indicated that 3-HAA has immunomodulatory roles that could play important roles in MS (184). This emerging evidence is discussed in Section “KP Influence on the Immune System” above.

## Oligodendrocytes as a Target Cell for Elevated Levels of KP Metabolites in MS

Oligodendrocytes are demyelinated and perish in MS most probably through the action of autoimmune T cells and associated neuroinflammation. As there is substantial influx of immune cells into the brain in MS, a significant body of evidence exists supporting a dysregulated KP in MS, which can favor the production of neurotoxic metabolites which further compromise oligodendrocyte health and function and could also contribute to neuronal atrophy. The known studies investigating the presence of KP metabolites and/or KP action on oligodendrocytes in MS are discussed below.

### Oligodendrocytes Express Only a Subset of KP Enzymes

Overall, while oligodendrocytes express several KP enzymes and may be able to uptake certain KP metabolites and participate in their metabolism, they appear not to express IDO-1, meaning that they are incapable of modulating T cell phenotype and KP action by metabolizing TRP. Radiolabeled QUIN was not detected in rat brain cultured oligodendrocytes after IFN-γ stimulation and incubation with radiolabeled TRP (185), leading to the subsequent conclusion following the study of human primary oligodendrocytes that oligodendrocytes are not capable of synthesizing QUIN *de novo*, rather they metabolize it (62). *Via* PCR, we demonstrated that IDO-1 is not expressed in human primary oligodendrocytes, even with IFN-γ stimulation (62), though data on adult oligodendrocytes are currently lacking. The enzymes tryptophan-2,3-dioxygenase (TDO) and kynurenine amino transferases II (KATII) were also not expressed, while KAT-I and enzymes further down the KP [KYNU, KMO, 3-hydroxyanthranilate oxygenase (3-HAO), and QPRT] were expressed, and increased in expression with IFN-γ stimulation.

The oligodendrocytes constitutively express the QPRT enzyme shows that they have the enzymatic machinery for further metabolism of QUIN to the essential cofactor NAD^+^ (62). QPRT was only modestly increased in expression in human oligodendrocytes in response to IFN-γ stimulation (62), suggesting that in inflammatory environments it could be easily saturated (considered further in Section “NMDA and Excitotoxicity”). Clearly, with regard to oligodendrocytes, the expression of QPRT and function in disease requires further study. Oligodendrocytes can also produce the neuroprotective PIC at concentrations of 45–55 nM [depending on whether IFN-γ stimulus is present (62)]; intriguingly, this was present at substantial (≈10-fold) excess compared with QUIN (even with IFN-γ stimulus) suggesting the predominant balance of the metabolism of the common QUIN/PIC precursor α-amino-β-carboxymuconate-ε-semialdehyde (ACMS) is skewed toward PIC production, or alternatively, that excess QUIN in oligodendrocytes is rapidly converted to NAD^+^ *via* QPRT. Our recent experiments in cultured oligodendrocytes [Ref. (20) and discussed in Section “Oligodendrocytes Are Particularly Sensitive to Quinolinic Acid Toxicity] currently do not support the latter hypothesis. The presence and function of 2-amino-3-carboxymuconate-6-semialdehyde decarboxylase (ACMSD) in oligodendrocytes is unknown at present, although from other studies its enzymatic activity is known to be partially inhibited by KYNA, QUIN, and PIC (186).

### Oligodendrocytes Are Particularly Sensitive to Quinolinic Acid Toxicity

Quinolinic acid is an NMDA receptor agonist and excitotoxin (139, 187), and oligodendrocytes are susceptible to NMDAR-mediated excitotoxicity with subsequent alterations to Ca^2+^ and other intracellular signaling that culminates in apoptosis (151, 152, 188–190). Therefore, in the context of activated monocyte influx during inflammatory demyelination episodes and secretion of QUIN, excitotoxicity induced by QUIN on oligodendrocytes is a major consideration in the progression of MS pathology. Conventional MS treatments can reduce the frequency of inflammatory episodes but do little to counteract the demyelination and death of oligodendrocytes (191).

Activated monocytes can enter the brain during BBB breakdown events and secrete QUIN or activate KP in neural cells by secretion of IFN-γ. Exposure of oligodendrocytes to excitotoxic QUIN at high levels, therefore, constitutes a further insult in concert with autoimmune-mediated demyelination. Indeed, we recently demonstrated by microscopy that QUIN can be rapidly taken up *in vitro* by cultured oligodendrocyte cell lines (substantial uptake was observed within 30–90 min), without significant degradation of QUIN. One possibility for the enhanced uptake is that the QPRT enzyme responsible for further metabolism of QUIN is saturated at a lower concentration, and hence in inflammatory circumstances QUIN accumulates in oligodendrocytes similar to that observed in neurons that are associated with Alzheimer’s pathology (168).

More importantly to MS pathology, these cells were also very sensitive to QUIN toxicity, which induced apoptotic cell death (LD50 0.5–1.0 μM in oligodendrocyte cell lines) (20). This is supported by the findings of others (151, 152). Apoptosis could be completely reversed by treatment with a monoclonal blocking antibody recognizing QUIN or by the use of specific IDO-1 enzyme inhibitors (to abolish QUIN production in monocyte-lineage cells) (20). In addition to QUIN concentration, chronicity of exposure and cell type is likely additional determinants of excitotoxic effects on brain cells. In the context of an inflammatory microenvironment, T-cell-induced damage to the myelin sheath as well as the toxicity induced by the presence of elevated levels of monocyte-produced QUIN are likely to significantly contribute to apoptosis of oligodendrocytes observed in MS.

Oligodendrocytes express multiple types of glutamate receptors, including *N*-methyl-d-aspartate (NMDA), alpha-amino-3-hydroxy-5-methylisoxazole-4-propionic acid (AMPA), and kainate. In white matter ischemic damage, excitotoxic cell death of oligodendrocytes *via* AMPA and kainate receptors are most often implicated (190, 192, 193). Oligodendrocytes lacking glutamate receptor subunit 2 (GluR2) are susceptible to AMPA/Kainate-induced excitotoxicity (190, 194). Spinal gray (195) and the myelinating processes of white matter oligodendrocytes express some NMDA receptors (188, 189, 196). Subsequent work confirmed that oligodendrocytes have an unusual NMDA receptor composition compared with neurons or other cells (197). Oligodendrocytes do express NR2A and 2B subunits (188), which have been confirmed in *Xenopus* oocytes to bind and be activated by QUIN, while NR2C subunits have 10-fold less affinity (198).

Although there is evidence that blockade of NMDA receptors is neuroprotective in inflammatory CNS diseases, and indeed can improve survival of oligodendrocytes (199, 200), future therapeutic use of this strategy needs to be tempered with an understanding that a “one-size-fits-all” approach such as broadly targeting NMDA receptors may not result in complete neurological disease resolution, and indeed, could have substantial side-effects including effects on other cell types such as neurons (201, 202). Hence, in our opinion, there is a clear notion that understanding the role of the KP in immune modulation and production of neurotoxic metabolites with action on oligodendrocytes could lead to the generation of alternative kinds of targeted therapies, which may be used as novel treatments for MS and possibly other autoimmune diseases.

## Modulation of the KP as a Therapeutic Strategy in MS

**Figure 6:**
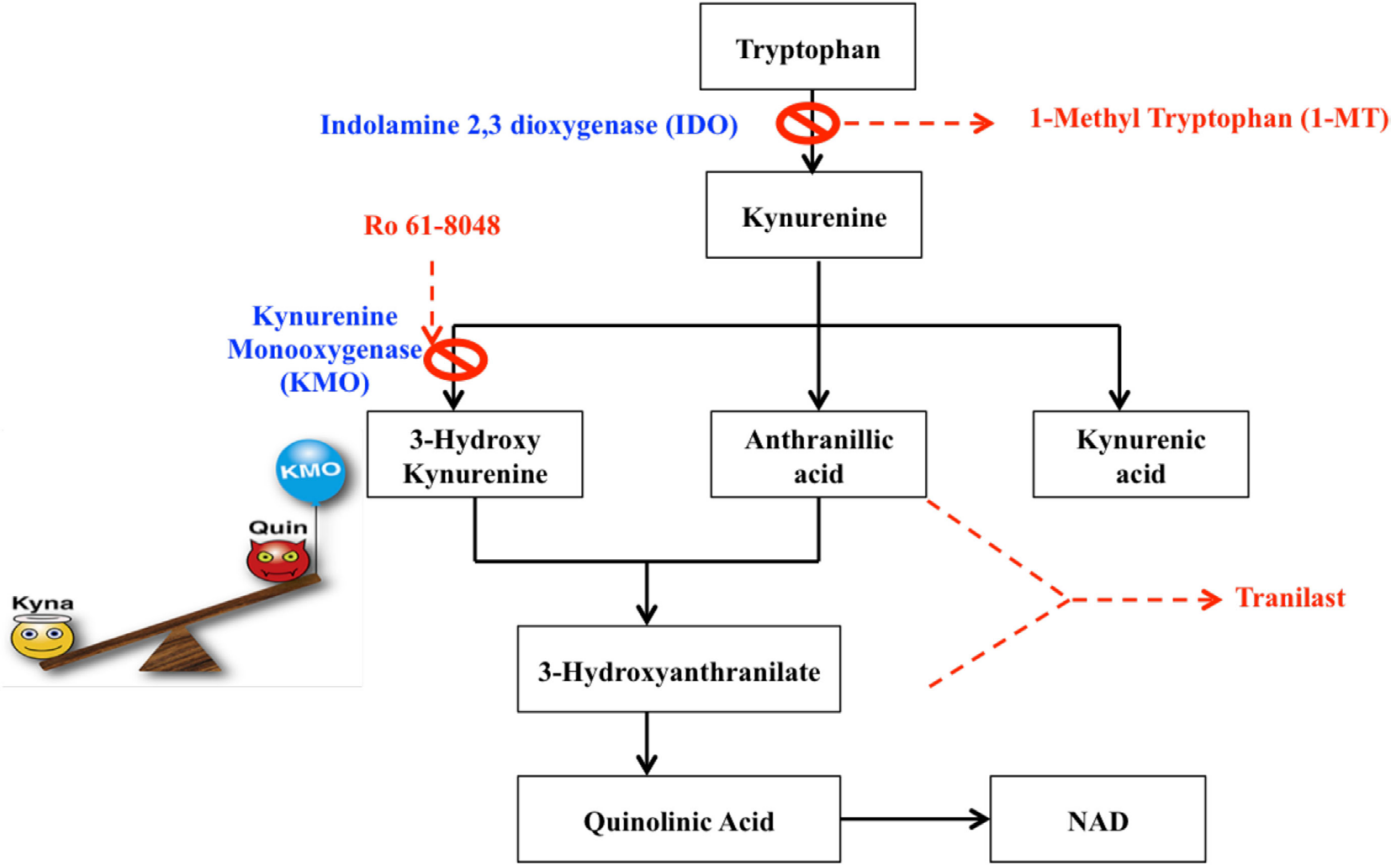
**Potential therapeutic targets for the modulation of the kynurenine pathway**. Dashed boxes indicate pharmacological analogs, whereas crosses indicate inhibitors.

Many therapeutic strategies are currently being pursued to protect against dysregulated KP metabolism in neurological diseases [summarized in Ref. (203)]. In the context of MS, current treatments are anti-inflammatory and although they effectively reduce the number and duration of relapses they do not appear capable of preventing long-term disability and mortality (204). This demonstrates an obvious lack of therapies that target the neurodegenerative components of the disease and that facilitate remyelination. As the KP is highly implicated in the pathophysiology of neurodegeneration and neuroinflammation, KP enzyme inhibitors together with kynurenine metabolites and their pharmacological analogs, could represent promising new therapeutic strategies that could target these mechanisms. A synthetic analog of the TRP metabolite *N*-[3,4-dimethoxycinnamoyl]-anthranilic acid (3,4-DAA), known commercially as Tranilast, has shown promising results in both *in vivo* and *in vitro* experiments (Figure 6).

Hertenstein et al. found that 3,4-DAA inhibited CD4^+^ T cell activation and proliferation and naive CD8^+^ T cells, although to a lesser extent (205). EAE animals treated with 3-HK or its synthetic derivate, Tranilast, showed reduced symptoms, number of relapses, and fewer inflammatory nodes in the spinal cord and brain (115, 206). In the EAE mouse model, the activity of KMO is significantly increased in spinal cords and this correlates with an increase in QUIN and 3-HK to neurotoxic levels. Administration of the KMO inhibitor Ro 61-8048 reduced the rise in levels of both QUIN and 3-HK, increased neuroprotective KYNA production and significantly alleviated disease progression (118). A number of immunomodulatory drugs such as leflunomide (the active metabolite is Teriflunomide) and Laquinimod, are structurally analogous to KYN and KYNA, respectively, and exhibit immunosuppressive properties by promoting a T_H_2 profile through a shift in cytokine balance and inhibiting activated T cells. These drugs were shown to ameliorate disease in EAE and Teriflunomide passed phase III clinical trials and is currently approved by the FDA. Although Laquinimod demonstrated only modest effects on the relapse rate in RRMS in phase III trials, it showed significant reductions in brain atrophy and is currently in phase III trials for PPMS, in which there are currently no approved disease-modifying treatments (207, 208). Together, the immunomodulatory and structural similarities of these drug candidates to kynurenines strongly implicate kynurenine analogs as novel and intriguing therapies for MS. The concept of KP therapies in MS is further supported by *in vitro* findings that QUIN is toxic to oligodendrocytes. Importantly, inhibiting IDO-1 (1-MT or berberine) or neutralizing QUIN directly with anti-QUIN antibodies overcame the toxicity toward oligodendrocytes that QUIN displayed (20).

Given that QUIN is present at pathophysiological concentrations that correlates with severity in EAE mice, manipulations that directly modulate its concentration-such as inhibition of IDO-1 or KMO are of considerable interest. However, systemic inhibition of IDO-1 has been demonstrated to exacerbate disease in EAE mice (18). This is due to the double nature of IDO-1 activation, where initial activation is immunosuppressive and is an important mechanism to counteract the autoimmune response; however, prolonged activation leads to the production of neurotoxic and oligotoxic kynurenines, thereby contributing to the pathology of MS. It is, therefore, likely that the timing of intervention is important. It is clear through both experimental and indirect evidence that the complex anti-inflammatory and neuroprotective properties of the KP metabolites have a fundamental link with MS. This warrants screening of these candidate drugs and highlights their therapeutic potential in MS.

## Microscope Imaging as a Central Tool for Advancing Knowledge of MS Pathology

**Figure 7:**
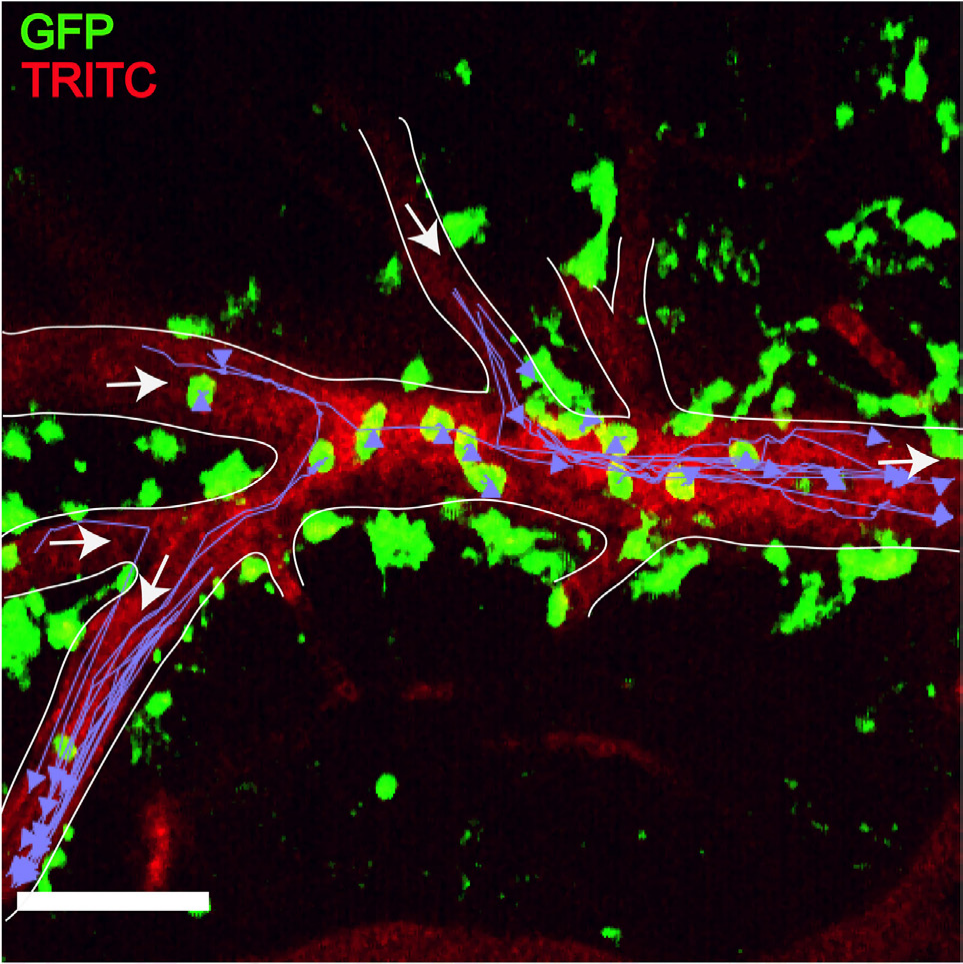
***Plasmodium*-primed CD8^+^ T cells induce monocyte accumulation in MacGreen × RAG^−/−^ mice**. PbA-infected MacGreen × RAG^−/−^ mice that had received primed CD8^+^ T cells underwent intravital imaging on day 7 p.i. (*n* = 3 mice/group). Representative snapshot shows severe levels of monocyte accumulation in the blood vessels. Scale bar represents 60 μm. Migratory paths of monocytes are shown as purple tracks. Blood vessels are marked by infusion of TRITC-conjugated dextran. White arrows indicate direction of blood flow. Data are a mean of two to three independent experiments [figure reproduced from Ref. (215), under Creative Commons Attribution license].

This review has discussed (particularly in Sections “The BBB Performs a Critical Role in Maintaining Barrier Integrity and Permeability, Which Is Lost in MS” and “Peripheral Blood Monocytes as a Potent Source of Inflammatory KP Metabolites in MS”) the significant achievements which have been generated by the use of imaging technology in MS studies, particularly non-invasive imaging such as MRI, and also correlative microscopy and histopathology. For mouse models of MS, multiphoton microscopy (Figure 7) has traditionally been the imaging technique of choice for imaging intact thick tissue sections (>200 μm) (209–212). Additionally, the easy availability of intravital imaging technology has greatly expanded the opportunity of directly observing the spatio-temporal context in which inflammatory events unfold *in situ* within the natural microenvironment of the CNS (213–215). Therefore, it is fast becoming the preferred modality for analyzing neuroinflammation *in vivo*. The advent of highly streamlined surgical protocols used in combination with two-photon intravital microscopy (2P-IVM) has proven to be powerful for characterizing the cellular and molecular mechanisms that underlie neuroinflammation (216–218). Its applicability across a breadth of neurological disciplines have helped identify novel routes of immune cell entry, their locomotion patterns, intravascular and transendothelial migration, homing, interactions with endothelial, immune, stromal, and neuronal cells, among others (219–223). These studies have significantly increased our understanding of the initiation and perpetuation of inflammation within the CNS and laid an excellent platform for making further advances within the field.

Our knowledge of how immune cells are recruited to the CNS; navigate through the CNS tissue; respond to self-antigens, pathogens, toxic metabolites, etc., and how they contribute to inflammatory diseases has been greatly informed by intravital imaging studies on EAE, one of the most intensely studied animal models of MS (224–228). In the past, EAE studies have been limited by the requirement for sophisticated imaging tools that can track T cell entry, behavior, and transmigration at cellular and subcellular resolution within the CNS. The complexities of tracking T cells in the CNS are related to the unique anatomical barriers such as the BBB that partly seclude the CNS from the constantly changing microenvironment of the blood stream, and its circulating immune cells (78, 229, 230). 2P-IVM (fluorochrome excited by two photons of 976 nm wavelength) circumvents some of these limitations due to its twin advantages of low phototoxicity and photobleaching, which enable long-term visualization (~1.5–6 h) and better penetrance (250–300 μm) of the CNS tissue (216).

Two-photon intravital microscopy studies have revealed that T_MBP_ cells (T cells recognizing oligodendrocyte MBP) first appear in the CNS vasculature during the build-up to EAE (225). Although it was not possible initially to ascertain the precise location of the infiltrating T_MBP_ cells using video microscopy, it is now clear that ~80% of the infiltrating T_MBP_ cells arrest and then crawl along the inner surface of the vessel wall soon after their entry (101). Only T_MBP_ cells that carry specific molecular signatures such as VLA-4 transmigrate through the BBB where they spend 80% of their time crawling once again on the abluminal surface of the vasculature, before detaching and crawling finally on the surface of the neuropil (224, 225). Therefore, infiltrating T_MBP_ cells crawl along three different cellular planes at different velocities with the end objective of “seeking” and contacting perivascular macrophages (PVM) that reside in the perivascular space (101, 231).

Real-time imaging studies have shown that after gaining passage into the perivascular space, T_MBP_ cells make short or long-lasting contact (>10 min) with PVM, antigen-presenting cells that strategically line blood vessels and monitor the environment with thin, motile cellular processes (209, 225, 232). Polarization of TCR and adhesion molecules such as LFA-1 as well as nuclear translocation of fluorescent NFAT, indicative of calcium-dependent T cell activation have been reported to occur at these contact points (227, 233). Interestingly, perivascular microglial and PVM clustering is one of the first pathological alterations that occurs in response to BBB disruption, during the build-up to EAE (234). As part of this alteration, cell body motility and process extension of microglia and PVM are specifically directed toward the affected vasculature where BBB disruption occurs. Thus, PVM clustering may only serve to enhance the chance of T_MBP_ cells encountering cognate antigen and invading the CNS parenchyma, besides its reported contribution to axonal damage.

Studies have shown that a majority of the T_MBP_ cells that invade the CNS parenchyma meander through the tissue (233). A small number exhibited confined motility, reminiscent of cells that have found cognate autoantigen. At first glance, these T_MBP_ cells appeared stationary; however, closer examination showed that they were remarkably agitated, rapidly extending and retracting cell protrusions and migrating at very low velocities through the tissue. Expectedly, extended recordings spanning several hours were necessary to resolve these fine locomotion patterns of T_MBP_ cells at the single cell level, especially since the sum of their directionality and length of migration remained close to zero (101, 233).

Despite the increasing number of real-time multiphoton studies that have yielded tremendous insights into the behavior of T cells in the processes leading to lesion formation at the single cell level in mouse MS models, application of this technology to the study of monocytes has yet to emerge. As described in Section “Peripheral Blood Monocytes as a Potent Source of Inflammatory KP Metabolites in MS,” evidence supports a role of activated monocytes in production of neurotoxic metabolites, which could contribute to MS pathology. Therefore, future studies to understand the behavior of monocytes as they pass into the brain parenchyma and interact closely with cells such as oligodendrocytes will provide additional insight to those gained recently by serial block-face reconstruction ultrastructure imaging (131).

## Summary and Conclusion

Imaging is now a central tool in MS research and treatment, allowing clinicians to evaluate the extent of the pathology and effectiveness of treatments while microscopy advances have allowed us to, in the case of multiphoton microscopy, peer deep into the brain to uncover mechanisms involved in immune cell trafficking and BBB dysfunction. Electron microscopy advances have also allowed automated processing and imaging of slices of tissue sections, allowing the ultrastructure of cell–cell interactions in MS to be confirmed. While MS is traditionally thought of in the context of aberrant autoimmune triggering of T cells, the multifaceted nature of the disease has emerged through increasing evidence of neurodegenerative pathology and the involvement of other cell types such as monocytes, and in turn signaling systems including the KP. This increasing recognition has led to the current notion of MS as involving a substantial degenerative disorder component (9). In this context, over the past two decades, a substantial body of literature has proven that dysregulation of the KP results in overproduction of neurotoxic metabolites that can potently kill brain cells. In MS, the extent of the pathology and disease progression correlates with elevated levels of neurotoxic metabolites which can contribute to the death of myelinating oligodendrocytes and their neighboring neurons, including during autoimmune inflammatory episodes. Therapeutic interventions to reduce the levels of damaging KP metabolites and improve oligodendrocyte and neuron survival are in continual development and progress toward clinical utility.

## Author Contributions

ML, BV, GS, NF, MN, SP, CL, GG, and BB – manuscript writing and final approval of manuscript. ML, BV, GS, SP, and NF – preparation of figures.

## Conflict of Interest Statement

The authors declare that the research was conducted in the absence of any commercial or financial relationships that could be construed as a potential conflict of interest.

## References

[1] Watzlawik JO Wootla B Rodriguez M. Tryptophan catabolites and their impact on multiple sclerosis progression. Curr Pharm Des (2016) 22:1049–59. 10.2174/1381612822666151215095940 26899126

[2] Dendrou CA Fugger L Friese MA. Immunopathology of multiple sclerosis. Nat Rev Immunol (2015) 15:545–58. 10.1038/nri3871 26250739

[3] Chard D Miller D. Grey matter pathology in clinically early multiple sclerosis: evidence from magnetic resonance imaging. J Neurol Sci (2009) 282:5–11. 10.1016/j.jns.2009.01.012 19201002

[4] Mitchell AJ Benito-Leon J Gonzalez JM Rivera-Navarro J. Quality of life and its assessment in multiple sclerosis: integrating physical and psychological components of wellbeing. Lancet Neurol (2005) 4:556–66. 10.1016/S1474-4422(05)70166-6 16109362

[5] Bashir K Whitaker JN Current immunotherapy for demyelinating diseases. Arch Neurol (2002) 59:726–31. 10.1001/archneur.59.5.726 12020252

[6] Goldenberg MM Multiple sclerosis review. P T (2012) 37:175–84. 22605909 PMC3351877

[7] Heyes MP Saito K Crowley JS Davis LE Demitrack MA Der M Quinolinic acid and kynurenine pathway metabolism in inflammatory and non-inflammatory neurological disease. Brain (1992) 115(Pt 5):1249–73. 10.1093/brain/115.5.1249 1422788

[8] Sanni LA Thomas SR Tattam BN Moore DE Chaudhri G Stocker R Dramatic changes in oxidative tryptophan metabolism along the kynurenine pathway in experimental cerebral and noncerebral malaria. Am J Pathol (1998) 152:611–9. 9466588 PMC1857979

[9] Stys PK Zamponi GW Van Minnen J Geurts JJ. Will the real multiple sclerosis please stand up? Nat Rev Neurosci (2012) 13:507–14. 10.1038/nrn3275 22714021

[10] Schwarcz R. The kynurenine pathway of tryptophan degradation as a drug target. Curr Opin Pharmacol (2004) 4:12–7. 10.1016/j.coph.2003.10.006 15018833

[11] Stone TW Darlington LG. The kynurenine pathway as a therapeutic target in cognitive and neurodegenerative disorders. Br J Pharmacol (2013) 169:1211–27. 10.1111/bph.12230 23647169 PMC3831703

[12] Jones SP Franco NF Varney B Sundaram G Brown DA De Bie J Expression of the kynurenine pathway in human peripheral blood mononuclear cells: implications for inflammatory and neurodegenerative disease. PLoS One (2015) 10:e0131389. 10.1371/journal.pone.0131389 26114426 PMC4482723

[13] Torok N Majlath Z Fulop F Toldi J Vecsei L. Brain aging and disorders of the central nervous system: kynurenines and drug metabolism. Curr Drug Metab (2016) 17:412–29. 10.2174/1389200217666151222155043 26694727

[14] Guillemin GJ Cullen KM Lim CK Smythe GA Garner B Kapoor V Characterization of the kynurenine pathway in human neurons. J Neurosci (2007) 27:12884–92. 10.1523/JNEUROSCI.4101-07.2007 18032661 PMC6673280

[15] Platten M Von Knebel Doeberitz N Oezen I Wick W Ochs K Cancer immunotherapy by targeting IDO1/TDO and their downstream effectors. Front Immunol (2014) 5:673 10.3389/fimmu.2014.00673 25628622 PMC4290671

[16] Ball HJ Sanchez-Perez A Weiser S Austin CJ Astelbauer F Miu J Characterization of an indoleamine 2,3-dioxygenase-like protein found in humans and mice. Gene (2007) 396:203–13. 10.1016/j.gene.2007.04.010 17499941

[17] Prendergast GC Metz R Muller AJ Merlo LM Mandik-Nayak L. IDO2 in immunomodulation and autoimmune disease. Front Immunol (2014) 5:585. 10.3389/fimmu.2014.00585 25477879 PMC4238401

[18] Vecsei L Szalardy L Fulop F Toldi J. Kynurenines in the CNS: recent advances and new questions. Nat Rev Drug Discov (2013) 12:64–82. 10.1038/nrd3793 23237916

[19] Chen Y Stankovic R Cullen KM Meininger V Garner B Coggan S The kynurenine pathway and inflammation in amyotrophic lateral sclerosis. Neurotox Res (2010) 18:132–42. 10.1007/s12640-009-9129-7 19921535

[20] Sundaram G Brew BJ Jones SP Adams S Lim CK Guillemin GJ. Quinolinic acid toxicity on oligodendroglial cells: relevance for multiple sclerosis and therapeutic strategies. J Neuroinflammation (2014) 11:204. 10.1186/s12974-014-0204-5 25498310 PMC4302518

[21] Guillemin GJ Kerr SJ Pemberton LA Smith DG Smythe GA Armati PJ IFN-beta1b induces kynurenine pathway metabolism in human macrophages: potential implications for multiple sclerosis treatment. J Interferon Cytokine Res (2001) 21:1097–101. 10.1089/107999001317205231 11798468

[22] Guillemin GJ Smith DG Kerr SJ Smythe GA Kapoor V Armati PJ Characterisation of kynurenine pathway metabolism in human astrocytes and implications in neuropathogenesis. Redox Rep (2000) 5:108–11. 10.1179/135100000101535375 10939285

[23] Guillemin GJ Smith DG Smythe GA Armati PJ Brew BJ Expression of the kynurenine pathway enzymes in human microglia and macrophages. Adv Exp Med Biol (2003) 527:105–12. 10.1007/978-1-4615-0135-0_12 15206722

[24] Guillemin GJ Smythe G Takikawa O Brew BJ Expression of indoleamine 2,3-dioxygenase and production of quinolinic acid by human microglia, astrocytes, and neurons. Glia (2004) 49:15–23. 10.1002/glia.20090 15390107

[25] Guillemin GJ Kerr SJ Smythe GA Smith DG Kapoor V Armati PJ Kynurenine pathway metabolism in human astrocytes: a paradox for neuronal protection. J Neurochem (2001) 78:842–53. 10.1046/j.1471-4159.2001.00498.x 11520905

[26] Lestage J Verrier D Palin K Dantzer R. The enzyme indoleamine 2,3-dioxygenase is induced in the mouse brain in response to peripheral administration of lipopolysaccharide and superantigen. Brain Behav Immun (2002) 16:596–601. 10.1016/S0889-1591(02)00014-4 12401474

[27] Hassanain HH Chon SY Gupta SL. Differential regulation of human indoleamine 2,3-dioxygenase gene expression by interferons-gamma and -alpha. Analysis of the regulatory region of the gene and identification of an interferon-gamma-inducible DNA-binding factor. J Biol Chem (1993) 268:5077–84. 8444884

[28] Babcock TA Carlin JM. Transcriptional activation of indoleamine dioxygenase by interleukin 1 and tumor necrosis factor alpha in interferon-treated epithelial cells. Cytokine (2000) 12:588–94. 10.1006/cyto.1999.0661 10843733

[29] Grohmann U Orabona C Fallarino F Vacca C Calcinaro F Falorni A CTLA-4-Ig regulates tryptophan catabolism in vivo. Nat Immunol (2002) 3:1097–101. 10.1038/ni846 12368911

[30] McIlroy D Tanguy-Royer S Le Meur N Guisle I Royer PJ Leger J Profiling dendritic cell maturation with dedicated microarrays. J Leukoc Biol (2005) 78:794–803. 10.1189/jlb.0105029 15961579

[31] Fujigaki S Saito K Sekikawa K Tone S Takikawa O Fujii H Lipopolysaccharide induction of indoleamine 2,3-dioxygenase is mediated dominantly by an IFN-gamma-independent mechanism. Eur J Immunol (2001) 31:2313–8. 10.1002/1521-4141(200108)31:8<2313::AID-IMMU2313>3.0.CO;2-S 11477543

[32] Hayashi T Beck L Rossetto C Gong X Takikawa O Takabayashi K Inhibition of experimental asthma by indoleamine 2,3-dioxygenase. J Clin Invest (2004) 114:270–9. 10.1172/JCI21275 15254594 PMC449749

[33] Hissong BD Carlin JM. Potentiation of interferon-induced indoleamine 2,3-dioxygenase mRNA in human mononuclear phagocytes by lipopolysaccharide and interleukin-1. J Interferon Cytokine Res (1997) 17:387–93. 10.1089/jir.1997.17.387 9243370

[34] Currier AR Ziegler MH Riley MM Babcock TA Telbis VP Carlin JM. Tumor necrosis factor-alpha and lipopolysaccharide enhance interferon-induced antichlamydial indoleamine dioxygenase activity independently. J Interferon Cytokine Res (2000) 20:369–76. 10.1089/107999000312306 10805371

[35] Robinson CM Shirey KA Carlin JM. Synergistic transcriptional activation of indoleamine dioxygenase by IFN-gamma and tumor necrosis factor-alpha. J Interferon Cytokine Res (2003) 23:413–21. 10.1089/107999003322277829 13678429 PMC1488822

[36] Pemberton LA Kerr SJ Smythe G Brew BJ. Quinolinic acid production by macrophages stimulated with IFN-gamma, TNF-alpha, and IFN-alpha. J Interferon Cytokine Res (1997) 17:589–95. 10.1089/jir.1997.17.589 9355959

[37] Alberati-Giani D Ricciardi-Castagnoli P Kohler C Cesura AM. Regulation of the kynurenine metabolic pathway by interferon-gamma in murine cloned macrophages and microglial cells. J Neurochem (1996) 66:996–1004. 10.1046/j.1471-4159.1996.66030996.x 8769859

[38] Heyes MP Chen CY Major EO Saito K. Different kynurenine pathway enzymes limit quinolinic acid formation by various human cell types. Biochem J (1997) 326(Pt 2):351–6. 10.1042/bj3260351 9291104 PMC1218677

[39] Heyes MP Saito K Lackner A Wiley CA Achim CL Markey SP. Sources of the neurotoxin quinolinic acid in the brain of HIV-1-infected patients and retrovirus-infected macaques. FASEB J (1998) 12:881–96. 9657528 10.1096/fasebj.12.10.881

[40] Zunszain PA Anacker C Cattaneo A Choudhury S Musaelyan K Myint AM Interleukin-1beta: a new regulator of the kynurenine pathway affecting human hippocampal neurogenesis. Neuropsychopharmacology (2012) 37:939–49. 10.1038/npp.2011.277 22071871 PMC3280640

[41] Bosco MC Rapisarda A Massazza S Melillo G Young H Varesio L. The tryptophan catabolite picolinic acid selectively induces the chemokines macrophage inflammatory protein-1 alpha and -1 beta in macrophages. J Immunol (2000) 164:3283–91. 10.4049/jimmunol.164.6.3283 10706721

[42] Melillo G Cox GW Radzioch D Varesio L. Picolinic acid, a catabolite of l-tryptophan, is a costimulus for the induction of reactive nitrogen intermediate production in murine macrophages. J Immunol (1993) 150:4031–40. 8473748

[43] Melillo G Cox GW Biragyn A Sheffler LA Varesio L. Regulation of nitric-oxide synthase mRNA expression by interferon-gamma and picolinic acid. J Biol Chem (1994) 269:8128–33. 7510678

[44] Law RO. Regulation of mammalian brain cell volume. J Exp Zool (1994) 268:90–6. 10.1002/jez.1402680204 8301256

[45] Simon MJ Iliff JJ Regulation of cerebrospinal fluid (CSF) flow in neurodegenerative, neurovascular and neuroinflammatory disease. Biochim Biophys Acta (2015) 1862(3):442–51. 10.1016/j.bbadis.2015.10.014 26499397 PMC4755861

[46] Weller RO. Pathology of cerebrospinal fluid and interstitial fluid of the CNS: significance for Alzheimer disease, prion disorders and multiple sclerosis. J Neuropathol Exp Neurol (1998) 57:885–94. 10.1097/00005072-199810000-00001 9786239

[47] Strazielle N Ghersi-Egea JF. Choroid plexus in the central nervous system: biology and physiopathology. J Neuropathol Exp Neurol (2000) 59:561–74. 10.1093/jnen/59.7.561 10901227

[48] Del Bigio MR. The ependyma: a protective barrier between brain and cerebrospinal fluid. Glia (1995) 14:1–13. 10.1002/glia.440140102 7615341

[49] Vigh-Teichmann I Vigh B. The cerebrospinal fluid-contacting neuron: a peculiar cell type of the central nervous system. Immunocytochemical aspects. Arch Histol Cytol (1989) 52(Suppl):195–207. 10.1679/aohc.52.Suppl_195 2479402

[50] Brinker T Stopa E Morrison J Klinge P. A new look at cerebrospinal fluid circulation. Fluids Barriers CNS (2014) 11:10. 10.1186/2045-8118-11-10 24817998 PMC4016637

[51] Marques F Sousa JC. The choroid plexus is modulated by various peripheral stimuli: implications to diseases of the central nervous system. Front Cell Neurosci (2015) 9:136. 10.3389/fncel.2015.00136 26236190 PMC4394702

[52] Kuenz B Lutterotti A Ehling R Gneiss C Haemmerle M Rainer C Cerebrospinal fluid B cells correlate with early brain inflammation in multiple sclerosis. PLoS One (2008) 3:e2559. 10.1371/journal.pone.0002559 18596942 PMC2438478

[53] Fritzsching B Haas J Konig F Kunz P Fritzsching E Poschl J Intracerebral human regulatory T cells: analysis of CD4+ CD25+ FOXP3+ T cells in brain lesions and cerebrospinal fluid of multiple sclerosis patients. PLoS One (2011) 6:e17988. 10.1371/journal.pone.0017988 21437244 PMC3060879

[54] Johanson CE Duncan JA Stopa EG Baird A. Enhanced prospects for drug delivery and brain targeting by the choroid plexus-CSF route. Pharm Res (2005) 22:1011–37. 10.1007/s11095-005-6039-0 16028003

[55] Reiber H Cerebrospinal fluid – physiology, analysis and interpretation of protein patterns for diagnosis of neurological diseases. Mult Scler (1998) 4:99–107. 10.1177/135245859800400302 9762655

[56] Cepok S Jacobsen M Schock S Omer B Jaekel S Boddeker I Patterns of cerebrospinal fluid pathology correlate with disease progression in multiple sclerosis. Brain (2001) 124:2169–76. 10.1093/brain/124.11.2169 11673319

[57] Guillemin G Brew BJ Noonan CE Knight TG Smythe G Cullen KM Mass spectrometric detection of quinolinic acid in microdissected Alzheimer’s disease plaques. Int Congr Ser (2007) 1304:404–8. 10.1016/j.ics.2007.07.012

[58] Chen Y Guillemin GJ. Kynurenine pathway metabolites in humans: disease and healthy states. Int J Tryptophan Res (2009) 2:1–19. 22084578 10.4137/ijtr.s2097 PMC3195227

[59] Guillemin GJ Williams KR Smith DG Smythe GA Croitoru-Lamoury J Brew BJ Quinolinic acid in the pathogenesis of Alzheimer’s disease. Adv Exp Med Biol (2003) 527:167–76. 10.1007/978-1-4615-0135-0_19 15206729

[60] Kerr SJ Armati PJ Guillemin GJ Brew BJ. Chronic exposure of human neurons to quinolinic acid results in neuronal changes consistent with AIDS dementia complex. AIDS (1998) 12:355–63. 10.1097/00002030-199804000-00003 9520164

[61] Kerr SJ Armati PJ Pemberton LA Smythe G Tattam B Brew BJ. Kynurenine pathway inhibition reduces neurotoxicity of HIV-1-infected macrophages. Neurology (1997) 49:1671–81. 10.1212/WNL.49.6.1671 9409365

[62] Lim CK Smythe G Stocker R Brew BJ Guillemin GJ Characterization of the kynurenine pathway in primary human oligodendrocytes. Int Congr Ser (2007) 1304:213–7. 10.1016/j.ics.2007.07.011

[63] Owe-Young R Webster NL Mukhtar M Pomerantz RJ Smythe G Walker D Kynurenine pathway metabolism in human blood-brain-barrier cells: implications for immune tolerance and neurotoxicity. J Neurochem (2008) 105:1346–57. 10.1111/j.1471-4159.2008.05241.x 18221377

[64] Komori M Blake A Greenwood M Lin YC Kosa P Ghazali D Cerebrospinal fluid markers reveal intrathecal inflammation in progressive multiple sclerosis. Ann Neurol (2015) 78:3–20. 10.1002/ana.24408 25808056 PMC5568079

[65] Villar LM Picon C Costa-Frossard L Alenda R Garcia-Caldentey J Espino M Cerebrospinal fluid immunological biomarkers associated with axonal damage in multiple sclerosis. Eur J Neurol (2015) 22:1169–75. 10.1111/ene.12579 25324032

[66] Modvig S Degn M Roed H Sorensen TL Larsson H Langkilde AR Cerebrospinal fluid levels of chitinase 3-like 1 and neurofilament light chain predict multiple sclerosis development and disability after optic neuritis. Mult Scler (2015) 21:1761–70. 10.1177/1352458515574148 25698172

[67] Flanagan EM Erickson JB Viveros OH Chang SY Reinhard JF Jr. Neurotoxin quinolinic acid is selectively elevated in spinal cords of rats with experimental allergic encephalomyelitis. J Neurochem (1995) 64:1192–6. 10.1046/j.1471-4159.1995.64031192.x 7861150

[68] Sakurai K Zou JP Tschetter JR Ward JM Shearer GM. Effect of indoleamine 2,3-dioxygenase on induction of experimental autoimmune encephalomyelitis. J Neuroimmunol (2002) 129:186–96. 10.1016/S0165-5728(02)00176-5 12161035

[69] Monaco F Fumero S Mondino A Mutani R. Plasma and cerebrospinal fluid tryptophan in multiple sclerosis and degenerative diseases. J Neurol Neurosurg Psychiatry (1979) 42:640–1. 10.1136/jnnp.42.7.640 479903 PMC490278

[70] Rejdak K Bartosik-Psujek H Dobosz B Kocki T Grieb P Giovannoni G Decreased level of kynurenic acid in cerebrospinal fluid of relapsing-onset multiple sclerosis patients. Neurosci Lett (2002) 331:63–5. 10.1016/S0304-3940(02)00710-3 12359324

[71] Rejdak K Petzold A Kocki T Kurzepa J Grieb P Turski WA Astrocytic activation in relation to inflammatory markers during clinical exacerbation of relapsing-remitting multiple sclerosis. J Neural Transm (Vienna) (2007) 114:1011–5. 10.1007/s00702-007-0667-y 17393066

[72] Hartai Z Klivenyi P Janaky T Penke B Dux L Vecsei L. Kynurenine metabolism in multiple sclerosis. Acta Neurol Scand (2005) 112:93–6. 10.1111/j.1600-0404.2005.00442.x 16008534

[73] Turski MP Turska M Paluszkiewicz P Parada-Turska J Oxenkrug GF. Kynurenic acid in the digestive system-new facts, new challenges. Int J Tryptophan Res (2013) 6:47–55. 10.4137/IJTR.S12536 24049450 PMC3772988

[74] Aeinehband S Brenner P Stahl S Bhat M Fidock MD Khademi M Cerebrospinal fluid kynurenines in multiple sclerosis; relation to disease course and neurocognitive symptoms. Brain Behav Immun (2016) 51:47–55. 10.1016/j.bbi.2015.07.016 26189678

[75] Hedegaard CJ Chen N Sellebjerg F Sorensen PS Leslie RG Bendtzen K Autoantibodies to myelin basic protein (MBP) in healthy individuals and in patients with multiple sclerosis: a role in regulating cytokine responses to MBP. Immunology (2009) 128:e451–61. 10.1111/j.1365-2567.2008.02999.x 19191913 PMC2753924

[76] Mancuso R Hernis A Agostini S Rovaris M Caputo D Fuchs D Indoleamine 2,3 dioxygenase (IDO) expression and activity in relapsing-remitting multiple sclerosis. PLoS One (2015) 10:e0130715. 10.1371/journal.pone.0130715 26110930 PMC4482492

[77] Sadowska-Bartosz I Adamczyk-Sowa M Gajewska A Bartosz G. Oxidative modification of blood serum proteins in multiple sclerosis after interferon or mitoxantrone treatment. J Neuroimmunol (2014) 266:67–74. 10.1016/j.jneuroim.2013.11.005 24290230

[78] Ransohoff RM Kivisakk P Kidd G. Three or more routes for leukocyte migration into the central nervous system. Nat Rev Immunol (2003) 3:569–81. 10.1038/nri1130 12876559

[79] Sedgwick JD Hughes CC Male DK Macphee IA Ter Meulen V. Antigen-specific damage to brain vascular endothelial cells mediated by encephalitogenic and nonencephalitogenic CD4+ T cell lines in vitro. J Immunol (1990) 145:2474–81. 1698855

[80] Hu P Pollard JD Chan-Ling T. Breakdown of the blood-retinal barrier induced by activated T cells of nonneural specificity. Am J Pathol (2000) 156:1139–49. 10.1016/S0002-9440(10)64982-6 10751337 PMC1876898

[81] Smorodchenko A Wuerfel J Pohl EE Vogt J Tysiak E Glumm R CNS-irrelevant T-cells enter the brain, cause blood-brain barrier disruption but no glial pathology. Eur J Neurosci (2007) 26:1387–98. 10.1111/j.1460-9568.2007.05792.x 17880383

[82] Larochelle C Alvarez JI Prat A. How do immune cells overcome the blood-brain barrier in multiple sclerosis? FEBS Lett (2011) 585:3770–80. 10.1016/j.febslet.2011.04.066 21550344

[83] Lopes Pinheiro MA Kooij G Mizee MR Kamermans A Enzmann G Lyck R Immune cell trafficking across the barriers of the central nervous system in multiple sclerosis and stroke. Biochim Biophys Acta (2015) 1862(3):461–71. 10.1016/j.bbadis.2015.10.018 26527183

[84] Hu P Pollard J Hunt N Chan-Ling T. Microvascular and cellular responses in the retina of rats with acute experimental allergic encephalomyelitis (EAE). Brain Pathol (1998) 8:487–98. 10.1111/j.1750-3639.1998.tb00170.x 9669699 PMC8098246

[85] Runge VM Schoerner W Niendorf HP Laniado M Koehler D Claussen C Initial clinical evaluation of gadolinium DTPA for contrast-enhanced magnetic resonance imaging. Magn Reson Imaging (1985) 3:27–35. 10.1016/0730-725X(85)90006-2 2987640

[86] Miller DH Rudge P Johnson G Kendall BE Macmanus DG Moseley IF Serial gadolinium enhanced magnetic resonance imaging in multiple sclerosis. Brain (1988) 111(Pt 4):927–39. 10.1093/brain/111.4.927 3401689

[87] Kermode AG Thompson AJ Tofts P Macmanus DG Kendall BE Kingsley DP Breakdown of the blood-brain barrier precedes symptoms and other MRI signs of new lesions in multiple sclerosis. Pathogenetic and clinical implications. Brain (1990) 113(Pt 5):1477–89. 10.1093/brain/113.5.1477 2245307

[88] Claudio L Kress Y Factor J Brosnan CF. Mechanisms of edema formation in experimental autoimmune encephalomyelitis. The contribution of inflammatory cells. Am J Pathol (1990) 137:1033–45. 2240157 PMC1877669

[89] Lossinsky AS Badmajew V Robson JA Moretz RC Wisniewski HM. Sites of egress of inflammatory cells and horseradish peroxidase transport across the blood-brain barrier in a murine model of chronic relapsing experimental allergic encephalomyelitis. Acta Neuropathol (1989) 78:359–71. 10.1007/BF00688172 2782047

[90] Goodkin DE Rooney WD Sloan R Bacchetti P Gee L Vermathen M A serial study of new MS lesions and the white matter from which they arise. Neurology (1998) 51:1689–97. 10.1212/WNL.51.6.1689 9855524

[91] Kirk J Plumb J Mirakhur M McQuaid S. Tight junctional abnormality in multiple sclerosis white matter affects all calibres of vessel and is associated with blood-brain barrier leakage and active demyelination. J Pathol (2003) 201:319–27. 10.1002/path.1434 14517850

[92] McQuaid S Cunnea P McMahon J Fitzgerald U. The effects of blood-brain barrier disruption on glial cell function in multiple sclerosis. Biochem Soc Trans (2009) 37:329–31. 10.1042/BST0370329 19143657

[93] Plumb J McQuaid S Mirakhur M Kirk J. Abnormal endothelial tight junctions in active lesions and normal-appearing white matter in multiple sclerosis. Brain Pathol (2002) 12:154–69. 10.1111/j.1750-3639.2002.tb00430.x 11958369 PMC8095734

[94] Vos CM Geurts JJ Montagne L Van Haastert ES Bo L Van Der Valk P Blood-brain barrier alterations in both focal and diffuse abnormalities on postmortem MRI in multiple sclerosis. Neurobiol Dis (2005) 20:953–60. 10.1016/j.nbd.2005.06.012 16039866

[95] Filippi M Rocca MA Martino G Horsfield MA Comi G. Magnetization transfer changes in the normal appearing white matter precede the appearance of enhancing lesions in patients with multiple sclerosis. Ann Neurol (1998) 43:809–14. 10.1002/ana.410430616 9629851

[96] Cramer SP Simonsen H Frederiksen JL Rostrup E Larsson HB Abnormal blood-brain barrier permeability in normal appearing white matter in multiple sclerosis investigated by MRI. Neuroimage Clin (2014) 4:182–9. 10.1016/j.nicl.2013.12.001 24371801 PMC3872721

[97] Ingrisch M Sourbron S Morhard D Ertl-Wagner B Kumpfel T Hohlfeld R Quantification of perfusion and permeability in multiple sclerosis: dynamic contrast-enhanced MRI in 3D at 3T. Invest Radiol (2012) 47:252–8. 10.1097/RLI.0b013e31823bfc97 22373532

[98] Atkins EJ Biousse V Newman NJ. The natural history of optic neuritis. Rev Neurol Dis (2006) 3:45–56. 16819420

[99] Cramer SP Modvig S Simonsen HJ Frederiksen JL Larsson HB. Permeability of the blood-brain barrier predicts conversion from optic neuritis to multiple sclerosis. Brain (2015) 138:2571–83. 10.1093/brain/awv203 26187333 PMC4547053

[100] Nathoo N Jalal H Natah S Zhang Q Wu Y Dunn J. Hypoxia and inflammation-induced disruptions of the blood-brain and blood-cerebrospinal fluid barriers assessed using a novel T1-based MRI method. Acta Neurochir Suppl (2016) 121:23–8. 10.1007/978-3-319-18497-5_5 26463918

[101] Kawakami N Flugel A Knocking at the brain’s door: intravital two-photon imaging of autoreactive T cell interactions with CNS structures. Semin Immunopathol (2010) 32:275–87. 10.1007/s00281-010-0216-x 20623286 PMC2937150

[102] Mandi Y Vecsei L The kynurenine system and immunoregulation. J Neural Transm (Vienna) (2012) 119:197–209. 10.1007/s00702-011-0681-y 21744051

[103] Bozza S Fallarino F Pitzurra L Zelante T Montagnoli C Bellocchio S A crucial role for tryptophan catabolism at the host/*Candida albicans* interface. J Immunol (2005) 174:2910–8. 10.4049/jimmunol.174.5.2910 15728502

[104] Munn DH Shafizadeh E Attwood JT Bondarev I Pashine A Mellor AL. Inhibition of T cell proliferation by macrophage tryptophan catabolism. J Exp Med (1999) 189:1363–72. 10.1084/jem.189.9.1363 10224276 PMC2193062

[105] Frumento G Rotondo R Tonetti M Damonte G Benatti U Ferrara GB. Tryptophan-derived catabolites are responsible for inhibition of T and natural killer cell proliferation induced by indoleamine 2,3-dioxygenase. J Exp Med (2002) 196:459–68. 10.1084/jem.20020121 12186838 PMC2196046

[106] Terness P Bauer TM Rose L Dufter C Watzlik A Simon H Inhibition of allogeneic T cell proliferation by indoleamine 2,3-dioxygenase-expressing dendritic cells: mediation of suppression by tryptophan metabolites. J Exp Med (2002) 196:447–57. 10.1084/jem.20020052 12186837 PMC2196057

[107] Fallarino F Grohmann U Vacca C Bianchi R Orabona C Spreca A T cell apoptosis by tryptophan catabolism. Cell Death Differ (2002) 9:1069–77. 10.1038/sj.cdd.4401073 12232795

[108] Bauer TM Jiga LP Chuang JJ Randazzo M Opelz G Terness P. Studying the immunosuppressive role of indoleamine 2,3-dioxygenase: tryptophan metabolites suppress rat allogeneic T-cell responses in vitro and in vivo. Transpl Int (2005) 18:95–100. 10.1111/j.1432-2277.2004.00031.x 15612990

[109] Della Chiesa M Carlomagno S Frumento G Balsamo M Cantoni C Conte R The tryptophan catabolite l-kynurenine inhibits the surface expression of NKp46- and NKG2D-activating receptors and regulates NK-cell function. Blood (2006) 108:4118–25. 10.1182/blood-2006-03-006700 16902152

[110] Fallarino F Grohmann U You S McGrath BC Cavener DR Vacca C The combined effects of tryptophan starvation and tryptophan catabolites down-regulate T cell receptor zeta-chain and induce a regulatory phenotype in naive T cells. J Immunol (2006) 176:6752–61. 10.4049/jimmunol.176.11.6752 16709834

[111] Pallotta MT Orabona C Volpi C Vacca C Belladonna ML Bianchi R Indoleamine 2,3-dioxygenase is a signaling protein in long-term tolerance by dendritic cells. Nat Immunol (2011) 12:870–8. 10.1038/ni.2077 21804557

[112] Belladonna ML Grohmann U Guidetti P Volpi C Bianchi R Fioretti MC Kynurenine pathway enzymes in dendritic cells initiate tolerogenesis in the absence of functional IDO. J Immunol (2006) 177:130–7. 10.4049/jimmunol.177.1.130 16785507

[113] Colin-Gonzalez AL Maldonado PD Santamaria A. 3-Hydroxykynurenine: an intriguing molecule exerting dual actions in the central nervous system. Neurotoxicology (2013) 34:189–204. 10.1016/j.neuro.2012.11.007 23219925

[114] Aranami T Yamamura T. Th17 cells and autoimmune encephalomyelitis (EAE/MS). Allergol Int (2008) 57:115–20. 10.2332/allergolint.R-07-159 18427164

[115] Yan Y Zhang GX Gran B Fallarino F Yu S Li H IDO upregulates regulatory T cells via tryptophan catabolite and suppresses encephalitogenic T cell responses in experimental autoimmune encephalomyelitis. J Immunol (2010) 185:5953–61. 10.4049/jimmunol.1001628 20944000 PMC2998795

[116] Kwidzinski E Bunse J Aktas O Richter D Mutlu L Zipp F Indolamine 2,3-dioxygenase is expressed in the CNS and down-regulates autoimmune inflammation. FASEB J (2005) 19:1347–9. 10.1096/fj.04-3228fje 15939737

[117] Xiao BG Wu XC Yang JS Xu LY Liu X Huang YM Therapeutic potential of IFN-gamma-modified dendritic cells in acute and chronic experimental allergic encephalomyelitis. Int Immunol (2004) 16:13–22. 10.1093/intimm/dxh003 14688056

[118] Chiarugi A Cozzi A Ballerini C Massacesi L Moroni F Kynurenine 3-mono-oxygenase activity and neurotoxic kynurenine metabolites increase in the spinal cord of rats with experimental allergic encephalomyelitis. Neuroscience (2001) 102:687–95. 10.1016/S0306-4522(00)00504-2 11226705

[119] Rajda C Majlath Z Pukoli D Vecsei L. Kynurenines and multiple sclerosis: the dialogue between the immune system and the central nervous system. Int J Mol Sci (2015) 16:18270–82. 10.3390/ijms160818270 26287161 PMC4581244

[120] Ott M Demisch L Engelhardt W Fischer PA. Interleukin-2, soluble interleukin-2-receptor, neopterin, l-tryptophan and beta 2-microglobulin levels in CSF and serum of patients with relapsing-remitting or chronic-progressive multiple sclerosis. J Neurol (1993) 241:108–14. 10.1007/BF00869773 8138825

[121] Kwidzinski E Bechmann I. IDO expression in the brain: a double-edged sword. J Mol Med (Berl) (2007) 85:1351–9. 10.1007/s00109-007-0229-7 17594069

[122] Auffray C Fogg D Garfa M Elain G Join-Lambert O Kayal S Monitoring of blood vessels and tissues by a population of monocytes with patrolling behavior. Science (2007) 317:666–70. 10.1126/science.1142883 17673663

[123] Mitchell AJ Roediger B Weninger W. Monocyte homeostasis and the plasticity of inflammatory monocytes. Cell Immunol (2014) 291:22–31. 10.1016/j.cellimm.2014.05.010 24962351

[124] King IL Dickendesher TL Segal BM. Circulating Ly-6C+ myeloid precursors migrate to the CNS and play a pathogenic role during autoimmune demyelinating disease. Blood (2009) 113:3190–7. 10.1182/blood-2008-07-168575 19196868 PMC2665891

[125] Ajami B Bennett JL Krieger C McNagny KM Rossi FM. Infiltrating monocytes trigger EAE progression, but do not contribute to the resident microglia pool. Nat Neurosci (2011) 14:1142–9. 10.1038/nn.2887 21804537

[126] Mishra MK Wang J Silva C Mack M Yong VW. Kinetics of proinflammatory monocytes in a model of multiple sclerosis and its perturbation by laquinimod. Am J Pathol (2012) 181:642–51. 10.1016/j.ajpath.2012.05.011 22749771

[127] Moreno MA Burns T Yao P Miers L Pleasure D Soulika AM. Therapeutic depletion of monocyte-derived cells protects from long-term axonal loss in experimental autoimmune encephalomyelitis. J Neuroimmunol (2016) 290:36–46. 10.1016/j.jneuroim.2015.11.004 26711567

[128] Ferguson B Matyszak MK Esiri MM Perry VH. Axonal damage in acute multiple sclerosis lesions. Brain (1997) 120(Pt 3):393–9. 10.1093/brain/120.3.393 9126051

[129] Trapp BD Peterson J Ransohoff RM Rudick R Mork S Bo L. Axonal transection in the lesions of multiple sclerosis. N Engl J Med (1998) 338:278–85. 10.1056/NEJM199801293380502 9445407

[130] Trebst C Sorensen TL Kivisakk P Cathcart MK Hesselgesser J Horuk R CCR1+/CCR5+ mononuclear phagocytes accumulate in the central nervous system of patients with multiple sclerosis. Am J Pathol (2001) 159:1701–10. 10.1016/S0002-9440(10)63017-9 11696431 PMC1867058

[131] Yamasaki R Lu H Butovsky O Ohno N Rietsch AM Cialic R Differential roles of microglia and monocytes in the inflamed central nervous system. J Exp Med (2014) 211:1533–49. 10.1084/jem.20132477 25002752 PMC4113947

[132] Braidy N Grant R Adams S Brew BJ Guillemin GJ Mechanism for quinolinic acid cytotoxicity in human astrocytes and neurons. Neurotox Res (2009) 16:77–86. 10.1007/s12640-009-9051-z 19526301

[133] Arellano G Ottum PA Reyes LI Burgos PI Naves R. Stage-specific role of interferon-gamma in experimental autoimmune encephalomyelitis and multiple sclerosis. Front Immunol (2015) 6:492. 10.3389/fimmu.2015.00492 26483787 PMC4586507

[134] Guillemin GJ Brew BJ Noonan CE Takikawa O Cullen KM Indoleamine 2,3 dioxygenase and quinolinic acid immunoreactivity in Alzheimer’s disease hippocampus. Neuropathol Appl Neurobiol (2005) 31:395–404. 10.1111/j.1365-2990.2005.00655.x 16008823

[135] Wu W Nicolazzo JA Wen L Chung R Stankovic R Bao SS Expression of tryptophan 2,3-dioxygenase and production of kynurenine pathway metabolites in triple transgenic mice and human Alzheimer’s disease brain. PLoS One (2013) 8:e59749 10.1371/journal.pone.0059749 23630570 PMC3632609

[136] Chen Y Brew BJ Guillemin GJ. Characterization of the kynurenine pathway in NSC-34 cell line: implications for amyotrophic lateral sclerosis. J Neurochem (2011) 118:816–25. 10.1111/j.1471-4159.2010.07159.x 21182524

[137] Schwarcz R Bruno JP Muchowski PJ Wu HQ. Kynurenines in the mammalian brain: when physiology meets pathology. Nat Rev Neurosci (2012) 13:465–77. 10.1038/nrn3257 22678511 PMC3681811

[138] Chiarugi A Meli E Moroni F Similarities and differences in the neuronal death processes activated by 3OH-kynurenine and quinolinic acid. J Neurochem (2001) 77:1310–8. 10.1046/j.1471-4159.2001.00335.x 11389182

[139] Guillemin GJ Quinolinic acid, the inescapable neurotoxin. FEBS J (2012) 279:1356–65. 10.1111/j.1742-4658.2012.08485.x 22248144

[140] Foster AC Okuno E Brougher DS Schwarcz R. A radioenzymatic assay for quinolinic acid. Anal Biochem (1986) 158:98–103. 10.1016/0003-2697(86)90595-6 2948416

[141] Guillemin GJ Quinolinic acid: neurotoxicity. FEBS J (2012) 279:1355 10.1111/j.1742-4658.2012.08493.x 22251552

[142] Braidy N Guillemin GJ Grant R. Effects of kynurenine pathway inhibition on NAD metabolism and cell viability in human primary astrocytes and neurons. Int J Tryptophan Res (2011) 4:29–37. 10.4137/IJTR.S7052 22084601 PMC3195218

[143] Lekieffre D Plotkine M Allix M Boulu RG. Kynurenic acid antagonizes hippocampal quinolinic acid neurotoxicity: behavioral and histological evaluation. Neurosci Lett (1990) 120:31–3. 10.1016/0304-3940(90)90160-B 2149878

[144] Sas K Robotka H Toldi J Vecsei L. Mitochondria, metabolic disturbances, oxidative stress and the kynurenine system, with focus on neurodegenerative disorders. J Neurol Sci (2007) 257:221–39. 10.1016/j.jns.2007.01.033 17462670

[145] Mehta A Prabhakar M Kumar P Deshmukh R Sharma PL. Excitotoxicity: bridge to various triggers in neurodegenerative disorders. Eur J Pharmacol (2013) 698:6–18. 10.1016/j.ejphar.2012.10.032 23123057

[146] Zhao C Deng W Gage FH. Mechanisms and functional implications of adult neurogenesis. Cell (2008) 132:645–60. 10.1016/j.cell.2008.01.033 18295581

[147] Croitoru-Lamoury J Lamoury FM Caristo M Suzuki K Walker D Takikawa O Interferon-gamma regulates the proliferation and differentiation of mesenchymal stem cells via activation of indoleamine 2,3 dioxygenase (IDO). PLoS One (2011) 6:e14698 10.1371/journal.pone.0014698 21359206 PMC3040184

[148] Grohmann U Bronte V. Control of immune response by amino acid metabolism. Immunol Rev (2010) 236:243–64. 10.1111/j.1600-065X.2010.00915.x 20636821

[149] Lugo-Huitron R Ugalde Muniz P Pineda B Pedraza-Chaverri J Rios C Perez-De La Cruz V. Quinolinic acid: an endogenous neurotoxin with multiple targets. Oxid Med Cell Longev (2013) 2013:104024. 10.1155/2013/104024 24089628 PMC3780648

[150] Sekine A Okamoto M Kanatani Y Sano M Shibata K Fukuwatari T. Amino acids inhibit kynurenic acid formation via suppression of kynurenine uptake or kynurenic acid synthesis in rat brain in vitro. Springerplus (2015) 4:48. 10.1186/s40064-015-0826-9 25674503 PMC4318830

[151] Cammer W. Oligodendrocyte killing by quinolinic acid in vitro. Brain Res (2001) 896:157–60. 10.1016/S0006-8993(01)02017-0 11277985

[152] Cammer W. Protection of cultured oligodendrocytes against tumor necrosis factor-alpha by the antioxidants coenzyme Q(10) and N-acetyl cysteine. Brain Res Bull (2002) 58:587–92. 10.1016/S0361-9230(02)00830-4 12372563

[153] Tavares RG Tasca CI Santos CE Wajner M Souza DO Dutra-Filho CS. Quinolinic acid inhibits glutamate uptake into synaptic vesicles from rat brain. Neuroreport (2000) 11:249–53. 10.1097/00001756-200002070-00005 10674464

[154] Tavares RG Tasca CI Santos CE Alves LB Porciuncula LO Emanuelli T Quinolinic acid stimulates synaptosomal glutamate release and inhibits glutamate uptake into astrocytes. Neurochem Int (2002) 40:621–7. 10.1016/S0197-0186(01)00133-4 11900857

[155] Piermartiri TC Vandresen-Filho S De Araujo Herculano B Martins WC Dal’Agnolo D Stroeh E Atorvastatin prevents hippocampal cell death due to quinolinic acid-induced seizures in mice by increasing AKT phosphorylation and glutamate uptake. Neurotox Res (2009) 16:106–15. 10.1007/s12640-009-9057-6 19526287

[156] Baverel G Martin G Michoudet C. Glutamine synthesis from aspartate in guinea-pig renal cortex. Biochem J (1990) 268:437–42. 10.1042/bj2680437 2363682 PMC1131451

[157] Ting KK Brew BJ Guillemin GJ Effect of quinolinic acid on human astrocytes morphology and functions: implications in Alzheimer’s disease. J Neuroinflammation (2009) 6:36 10.1186/1742-2094-6-36 20003262 PMC2797503

[158] Goda K Kishimoto R Shimizu S Hamane Y Ueda M Quinolinic acid and active oxygens. Possible contribution of active oxygens during cell death in the brain. Adv Exp Med Biol (1996) 398:247–54. 10.1007/978-1-4613-0381-7_38 8906272

[159] Muller AC Dairam A Limson JL Daya S. Mechanisms by which acyclovir reduces the oxidative neurotoxicity and biosynthesis of quinolinic acid. Life Sci (2007) 80:918–25. 10.1016/j.lfs.2006.11.031 17174341

[160] Rodriguez-Martinez E Camacho A Maldonado PD Pedraza-Chaverri J Santamaria D Galvan-Arzate S Effect of quinolinic acid on endogenous antioxidants in rat corpus striatum. Brain Res (2000) 858:436–9. 10.1016/S0006-8993(99)02474-9 10708698

[161] Tasset I Perez-De La Cruz V Elinos-Calderon D Carrillo-Mora P Gonzalez-Herrera IG Luna-Lopez A Protective effect of tert-butylhydroquinone on the quinolinic-acid-induced toxicity in rat striatal slices: role of the NRF2-antioxidant response element pathway. Neurosignals (2010) 18:24–31. 10.1159/000243650 19797933

[162] Franklin RJ Ffrench-Constant C Edgar JM Smith KJ. Neuroprotection and repair in multiple sclerosis. Nat Rev Neurol (2012) 8:624–34. 10.1038/nrneurol.2012.200 23026979

[163] Nikic I Merkler D Sorbara C Brinkoetter M Kreutzfeldt M Bareyre FM A reversible form of axon damage in experimental autoimmune encephalomyelitis and multiple sclerosis. Nat Med (2011) 17:495–9. 10.1038/nm.2324 21441916

[164] Haider L Fischer MT Frischer JM Bauer J Hoftberger R Botond G Oxidative damage in multiple sclerosis lesions. Brain (2011) 134:1914–24. 10.1093/brain/awr128 21653539 PMC3122372

[165] Kubicova L Hadacek F Chobot V. Quinolinic acid: neurotoxin or oxidative stress modulator? Int J Mol Sci (2013) 14:21328–38. 10.3390/ijms141121328 24232578 PMC3856007

[166] Kubicova L Hadacek F Weckwerth W Chobot V. Effects of endogenous neurotoxin quinolinic acid on reactive oxygen species production by Fenton reaction catalyzed by iron or copper. J Organomet Chem (2015) 782:111–5. 10.1016/j.jorganchem.2015.01.030 25892824 PMC4396856

[167] Pierozan P Zamoner A Soska AK Silvestrin RB Loureiro SO Heimfarth L Acute intrastriatal administration of quinolinic acid provokes hyperphosphorylation of cytoskeletal intermediate filament proteins in astrocytes and neurons of rats. Exp Neurol (2010) 224:188–96. 10.1016/j.expneurol.2010.03.009 20303347

[168] Rahman A Ting K Cullen KM Braidy N Brew BJ Guillemin GJ. The excitotoxin quinolinic acid induces tau phosphorylation in human neurons. PLoS One (2009) 4:e6344. 10.1371/journal.pone.0006344 19623258 PMC2709912

[169] Anderson JM Hampton DW Patani R Pryce G Crowther RA Reynolds R Abnormally phosphorylated tau is associated with neuronal and axonal loss in experimental autoimmune encephalomyelitis and multiple sclerosis. Brain (2008) 131:1736–48. 10.1093/brain/awn119 18567922

[170] Pierozan P Ferreira F Ortiz De Lima B Goncalves Fernandes C Totarelli Monteforte P De Castro Medaglia N The phosphorylation status and cytoskeletal remodeling of striatal astrocytes treated with quinolinic acid. Exp Cell Res (2014) 322:313–23. 10.1016/j.yexcr.2014.02.024 24583400

[171] Pierozan P Ferreira F De Lima BO Pessoa-Pureur R. Quinolinic acid induces disrupts cytoskeletal homeostasis in striatal neurons. Protective role of astrocyte-neuron interaction. J Neurosci Res (2015) 93:268–84. 10.1002/jnr.23494 25306914

[172] Fernandes AM Landeira-Fernandez AM Souza-Santos P Carvalho-Alves PC Castilho RF. Quinolinate-induced rat striatal excitotoxicity impairs endoplasmic reticulum Ca2+-ATPase function. Neurochem Res (2008) 33:1749–58. 10.1007/s11064-008-9619-7 18307036

[173] Vamos E Pardutz A Klivenyi P Toldi J Vecsei L. The role of kynurenines in disorders of the central nervous system: possibilities for neuroprotection. J Neurol Sci (2009) 283:21–7. 10.1016/j.jns.2009.02.326 19268309

[174] Okuda S Nishiyama N Saito H Katsuki H. Hydrogen peroxide-mediated neuronal cell death induced by an endogenous neurotoxin, 3-hydroxykynurenine. Proc Natl Acad Sci U S A (1996) 93:12553–8. 10.1073/pnas.93.22.12553 8901620 PMC38030

[175] Okuda S Nishiyama N Saito H Katsuki H. 3-Hydroxykynurenine, an endogenous oxidative stress generator, causes neuronal cell death with apoptotic features and region selectivity. J Neurochem (1998) 70:299–307. 10.1046/j.1471-4159.1998.70010299.x 9422375

[176] Smith AJ Smith RA Stone TW. 5-Hydroxyanthranilic acid, a tryptophan metabolite, generates oxidative stress and neuronal death via p38 activation in cultured cerebellar granule neurones. Neurotox Res (2009) 15:303–10. 10.1007/s12640-009-9034-0 19384564

[177] Jeong JH Kim HJ Lee TJ Kim MK Park ES Choi BS. Epigallocatechin 3-gallate attenuates neuronal damage induced by 3-hydroxykynurenine. Toxicology (2004) 195:53–60. 10.1016/j.tox.2003.08.007 14698567

[178] Braidy N Grant R Brew BJ Adams S Jayasena T Guillemin GJ Effects of kynurenine pathway metabolites on intracellular NAD synthesis and cell death in human primary astrocytes and neurons. Int J Tryptophan Res (2009) 2:61–9. 22084582 10.4137/ijtr.s2318 PMC3195228

[179] Berger F Ramirez-Hernandez MH Ziegler M. The new life of a centenarian: signalling functions of NAD(P). Trends Biochem Sci (2004) 29:111–8. 10.1016/j.tibs.2004.01.007 15003268

[180] Zhang T Berrocal JG Frizzell KM Gamble MJ Dumond ME Krishnakumar R Enzymes in the NAD+ salvage pathway regulate SIRT1 activity at target gene promoters. J Biol Chem (2009) 284:20408–17. 10.1074/jbc.M109.016469 19478080 PMC2740465

[181] Guidetti P Schwarcz R. 3-Hydroxykynurenine potentiates quinolinate but not NMDA toxicity in the rat striatum. Eur J Neurosci (1999) 11:3857–63. 10.1046/j.1460-9568.1999.00806.x 10583474

[182] Schwarcz R Guidetti P Sathyasaikumar KV Muchowski PJ Of mice, rats and men: revisiting the quinolinic acid hypothesis of Huntington’s disease. Prog Neurobiol (2010) 90:230–45. 10.1016/j.pneurobio.2009.04.005 19394403 PMC2829333

[183] Stone TW Darlington LG. Endogenous kynurenines as targets for drug discovery and development. Nat Rev Drug Discov (2002) 1:609–20. 10.1038/nrd870 12402501

[184] Darlington LG Forrest CM Mackay GM Smith RA Smith AJ Stoy N On the biological importance of the 3-hydroxyanthranilic acid: anthranilic acid ratio. Int J Tryptophan Res (2010) 3:51–9. 10.4137/IJTR.S4282 22084587 PMC3195249

[185] Saito K Chen CY Masana M Crowley JS Markey SP Heyes MP. 4-Chloro-3-hydroxyanthranilate, 6-chlorotryptophan and norharmane attenuate quinolinic acid formation by interferon-gamma-stimulated monocytes (THP-1 cells). Biochem J (1993) 291(Pt 1):11–4. 10.1042/bj2910011 8471029 PMC1132472

[186] Pucci L Perozzi S Cimadamore F Orsomando G Raffaelli N. Tissue expression and biochemical characterization of human 2-amino 3-carboxymuconate 6-semialdehyde decarboxylase, a key enzyme in tryptophan catabolism. FEBS J (2007) 274:827–40. 10.1111/j.1742-4658.2007.05635.x 17288562

[187] Stone TW Perkins MN Quinolinic acid: a potent endogenous excitant at amino acid receptors in CNS. Eur J Pharmacol (1981) 72:411–2. 10.1016/0014-2999(81)90587-2 6268428

[188] Salter MG Fern R. NMDA receptors are expressed in developing oligodendrocyte processes and mediate injury. Nature (2005) 438:1167–71. 10.1038/nature04301 16372012

[189] Micu I Jiang Q Coderre E Ridsdale A Zhang L Woulfe J NMDA receptors mediate calcium accumulation in myelin during chemical ischaemia. Nature (2006) 439:988–92. 10.1038/nature04474 16372019

[190] Matute C Alberdi E Domercq M Perez-Cerda F Perez-Samartin A Sanchez-Gomez MV. The link between excitotoxic oligodendroglial death and demyelinating diseases. Trends Neurosci (2001) 24:224–30. 10.1016/S0166-2236(00)01746-X 11250007

[191] Williams A. Remyelination in multiple sclerosis: what do we know and where are we going? Neurodegener Dis Manag (2015) 5:49–59. 10.2217/nmt.14.40 25711454

[192] Matute C Alberdi E Domercq M Sanchez-Gomez MV Perez-Samartin A Rodriguez-Antiguedad A Excitotoxic damage to white matter. J Anat (2007) 210:693–702. 10.1111/j.1469-7580.2007.00733.x 17504270 PMC2375761

[193] Sanchez-Gomez MV Alberdi E Ibarretxe G Torre I Matute C. Caspase-dependent and caspase-independent oligodendrocyte death mediated by AMPA and kainate receptors. J Neurosci (2003) 23:9519–28. 14573531 10.1523/JNEUROSCI.23-29-09519.2003 PMC6740470

[194] McDonald JW Levine JM Qu Y. Multiple classes of the oligodendrocyte lineage are highly vulnerable to excitotoxicity. Neuroreport (1998) 9:2757–62. 10.1097/00001756-199808240-00014 9760116

[195] Ziak D Chvatal A Sykova E. Glutamate-, kainate- and NMDA-evoked membrane currents in identified glial cells in rat spinal cord slice. Physiol Res (1998) 47:365–75. 10052606

[196] Karadottir R Cavelier P Bergersen LH Attwell D. NMDA receptors are expressed in oligodendrocytes and activated in ischaemia. Nature (2005) 438:1162–6. 10.1038/nature04302 16372011 PMC1416283

[197] Bakiri Y Hamilton NB Karadottir R Attwell D. Testing NMDA receptor block as a therapeutic strategy for reducing ischaemic damage to CNS white matter. Glia (2008) 56:233–40. 10.1002/glia.20608 18046734 PMC2863073

[198] de Carvalho LP Bochet P Rossier J. The endogenous agonist quinolinic acid and the non endogenous homoquinolinic acid discriminate between NMDAR2 receptor subunits. Neurochem Int (1996) 28:445–52. 10.1016/0197-0186(95)00091-7 8740453

[199] Pitt D Werner P Raine CS. Glutamate excitotoxicity in a model of multiple sclerosis. Nat Med (2000) 6:67–70. 10.1038/71555 10613826

[200] Smith T Groom A Zhu B Turski L. Autoimmune encephalomyelitis ameliorated by AMPA antagonists. Nat Med (2000) 6:62–6. 10.1038/71548 10613825

[201] Ikonomidou C Turski L. Why did NMDA receptor antagonists fail clinical trials for stroke and traumatic brain injury? Lancet Neurol (2002) 1:383–6. 10.1016/S1474-4422(02)00164-3 12849400

[202] Muir KW. Glutamate-based therapeutic approaches: clinical trials with NMDA antagonists. Curr Opin Pharmacol (2006) 6:53–60. 10.1016/j.coph.2005.12.002 16359918

[203] Lovelace MD Varney B Sundaram G Lennon MJ Lim CK Jacobs K Recent evidence for an expanded role of the kynurenine pathway of tryptophan metabolism in neurological diseases. Neuropharmacology (2016). 10.1016/j.neuropharm.2016.03.024 26995730

[204] Haghikia A Hohlfeld R Gold R Fugger L. Therapies for multiple sclerosis: translational achievements and outstanding needs. Trends Mol Med (2013) 19:309–19. 10.1016/j.molmed.2013.03.004 23582699

[205] Hertenstein A Schumacher T Litzenburger U Opitz CA Falk CS Serafini T Suppression of human CD4+ T cell activation by 3,4-dimethoxycinnamonyl-anthranilic acid (tranilast) is mediated by CXCL9 and CXCL10. Biochem Pharmacol (2011) 82:632–41. 10.1016/j.bcp.2011.06.013 21703247

[206] Platten M Ho PP Youssef S Fontoura P Garren H Hur EM Treatment of autoimmune neuroinflammation with a synthetic tryptophan metabolite. Science (2005) 310:850–5. 10.1126/science.1117634 16272121

[207] Constantinescu SE Constantinescu CS. Laquinimod (ABR-215062) for the treatment of relapsing multiple sclerosis. Expert Rev Clin Pharmacol (2016) 9:49–57. 10.1586/17512433.2016.1108189 26536299

[208] Vollmer TL Sorensen PS Selmaj K Zipp F Havrdova E Cohen JA A randomized placebo-controlled phase III trial of oral laquinimod for multiple sclerosis. J Neurol (2014) 261:773–83. 10.1007/s00415-014-7264-4 24535134

[209] Abtin A Jain R Mitchell AJ Roediger B Brzoska AJ Tikoo S Perivascular macrophages mediate neutrophil recruitment during bacterial skin infection. Nat Immunol (2014) 15:45–53. 10.1038/ni.2769 24270515 PMC4097073

[210] Roediger B Kyle R Yip KH Sumaria N Guy TV Kim BS Cutaneous immunosurveillance and regulation of inflammation by group 2 innate lymphoid cells. Nat Immunol (2013) 14:564–73. 10.1038/ni.2584 23603794 PMC4282745

[211] Li JL Goh CC Keeble JL Qin JS Roediger B Jain R Intravital multiphoton imaging of immune responses in the mouse ear skin. Nat Protoc (2012) 7:221–34. 10.1038/nprot.2011.438 22240584

[212] Roediger B Ng LG Smith AL Fazekas De St Groth B Weninger W. Visualizing dendritic cell migration within the skin. Histochem Cell Biol (2008) 130:1131–46. 10.1007/s00418-008-0531-7 18987873

[213] Germain RN Miller MJ Dustin ML Nussenzweig MC. Dynamic imaging of the immune system: progress, pitfalls and promise. Nat Rev Immunol (2006) 6:497–507. 10.1038/nri1884 16799470

[214] Padmanabhan K Andrews SE Fitzpatrick JA. Multi-photon imaging. Curr Protoc Cytom (2010) Chapter 2:Unit 2.9. 10.1002/0471142956.cy0209s54 20938919

[215] Pai S Qin J Cavanagh L Mitchell A El-Assaad F Jain R Real-time imaging reveals the dynamics of leukocyte behaviour during experimental cerebral malaria pathogenesis. PLoS Pathog (2014) 10:e1004236. 10.1371/journal.ppat.1004236 25033406 PMC4102563

[216] Pai S Danne KJ Qin J Cavanagh LL Smith A Hickey MJ Visualizing leukocyte trafficking in the living brain with 2-photon intravital microscopy. Front Cell Neurosci (2012) 6:67 10.3389/fncel.2012.00067 23316136 PMC3539661

[217] Cabrales P Carvalho LJM. Intravital microscopy of the mouse brain microcirculation using a closed cranial window. J Vis Exp (2010) 45:e2184. 10.3791/2184 21113121 PMC3074458

[218] Holtmaat A Bonhoeffer T Chow DK Chuckowree J De Paola V Hofer SB Long-term, high-resolution imaging in the mouse neocortex through a chronic cranial window. Nat Protoc (2009) 4:1128–44. 10.1038/nprot.2009.89 19617885 PMC3072839

[219] Fabene PF Navarro Mora G Martinello M Rossi B Merigo F Ottoboni L A role for leukocyte-endothelial adhesion mechanisms in epilepsy. Nat Med (2008) 14:1377–83. 10.1038/nm.1878 19029985 PMC2710311

[220] Wilson EH Weninger W Hunter CA. Trafficking of immune cells in the central nervous system. J Clin Invest (2010) 120:1368–79. 10.1172/JCI41911 20440079 PMC2860945

[221] Mrass P Weninger W. Immune cell migration as a means to control immune privilege: lessons from the CNS and tumors. Immunol Rev (2006) 213:195–212. 10.1111/j.1600-065X.2006.00433.x 16972905

[222] Harris TH Banigan EJ Christian DA Konradt C Tait Wojno ED Norose K Generalized Levy walks and the role of chemokines in migration of effector CD8+ T cells. Nature (2012) 486:545–8. 10.1038/nature11098 22722867 PMC3387349

[223] McGavern DB Kang SS. Illuminating viral infections in the nervous system. Nat Rev Immunol (2011) 11:318–29. 10.1038/nri2971 21508982 PMC5001841

[224] Siffrin V Brandt AU Radbruch H Herz J Boldakowa N Leuenberger T Differential immune cell dynamics in the CNS cause CD4+ T cell compartmentalization. Brain (2009) 132:1247–58. 10.1093/brain/awn354 19179377

[225] Bartholomaus I Kawakami N Odoardi F Schlager C Miljkovic D Ellwart JW Effector T cell interactions with meningeal vascular structures in nascent autoimmune CNS lesions. Nature (2009) 462:94–8. 10.1038/nature08478 19829296

[226] Constantin G Marconi S Rossi B Angiari S Calderan L Anghileri E Adipose-derived mesenchymal stem cells ameliorate chronic experimental autoimmune encephalomyelitis. Stem Cells (2009) 27:2624–35. 10.1002/stem.194 19676124

[227] Pesic M Bartholomaus I Kyratsous NI Heissmeyer V Wekerle H Kawakami N. 2-Photon imaging of phagocyte-mediated T cell activation in the CNS. J Clin Invest (2013) 123:1192–201. 10.1172/JCI67233 23454769 PMC3582148

[228] Siffrin V Radbruch H Glumm R Niesner R Paterka M Herz J In vivo imaging of partially reversible th17 cell-induced neuronal dysfunction in the course of encephalomyelitis. Immunity (2010) 33:424–36. 10.1016/j.immuni.2010.08.018 20870176

[229] Engelhardt B Ransohoff RM. The ins and outs of T-lymphocyte trafficking to the CNS: anatomical sites and molecular mechanisms. Trends Immunol (2005) 26:485–95. 10.1016/j.it.2005.07.004 16039904

[230] Ransohoff RM Engelhardt B. The anatomical and cellular basis of immune surveillance in the central nervous system. Nat Rev Immunol (2012) 12:623–35. 10.1038/nri3265 22903150

[231] Owens T Bechmann I Engelhardt B. Perivascular spaces and the two steps to neuroinflammation. J Neuropathol Exp Neurol (2008) 67:1113–21. 10.1097/NEN.0b013e31818f9ca8 19018243

[232] Nayak D Johnson KR Heydari S Roth TL Zinselmeyer BH McGavern DB. Type I interferon programs innate myeloid dynamics and gene expression in the virally infected nervous system. PLoS Pathog (2013) 9:e1003395. 10.1371/journal.ppat.1003395 23737750 PMC3667771

[233] Kawakami N Nagerl UV Odoardi F Bonhoeffer T Wekerle H Flugel A. Live imaging of effector cell trafficking and autoantigen recognition within the unfolding autoimmune encephalomyelitis lesion. J Exp Med (2005) 201:1805–14. 10.1084/jem.20050011 15939794 PMC2213265

[234] Davalos D Ryu JK Merlini M Baeten KM Le Moan N Petersen MA Fibrinogen-induced perivascular microglial clustering is required for the development of axonal damage in neuroinflammation. Nat Commun (2012) 3:1227. 10.1038/ncomms2230 23187627 PMC3514498

[235] O’Connor PW Li D Freedman MS Bar-Or A Rice GP Confavreux C A phase II study of the safety and efficacy of teriflunomide in multiple sclerosis with relapses. Neurology (2006) 66:894–900. 10.1212/01.wnl.0000203121.04509.31 16567708

[236] Chen Y Guillemin G The kynurenine pathway. In: Maurer M, editor. Amyotrophic Lateral Sclerosis, Chapter 15. InTech (2012). Available from: http://www.intechopen.com/books/amyotrophic-lateral-sclerosis/the-kynurenine-pathway

